# Orientation tomography of lanthanide-doped nanoprobes by polarization-resolved micro-spectroscopy

**DOI:** 10.1038/s41467-026-78323-0

**Published:** 2026-10-14

**Authors:** Lilian Magermans, Marcello Bonetti, Thierry Gacoin, Jongwook Kim

**Affiliations:** Laboratoire de Physique de la Matière Condensée – CNRS, Ecole Polytechnique, Institut Polytechnique de Paris, Palaiseau, France

**Keywords:** Nanoparticles, Imaging and sensing, Colloids

## Abstract

Quantifying three-dimensional (3D) orientation is critical for understanding self-assembly and collective behavior in soft matter and biological systems. Moreover, orientational analysis of anisotropic particles can characterize locally applied forces in fluids. We present a methodology for 3D orientation tomography based on polarization-resolved spectroscopy of lanthanide-doped nanorods exhibiting multiple emission dipoles, enabling 3D orientation measurements from a single viewing angle. We develop a confocal imaging setup optimized for lanthanide luminescence and a theoretical framework to extract both the local director and order parameter in nanorod assemblies. The technique achieves 0.5 μm lateral and 1 μm axial spatial resolution and is demonstrated in dense nematic liquid-crystal assemblies, resolving complex 3D topological structures, and in microfluidic flows, mapping alignment under hydrodynamic shear. This approach provides a versatile platform for quantitative studies of collective orientation, self-assembly mechanics, and alignment induced by external forces, and can be extended to other lanthanide-labeled particles and molecular probes.

## Abstract (web summary)

A new spectroscopic method for tomographic mapping of the 3D orientation of nanocrystals reveals complex structures and dynamics in both spontaneously formed and externally driven assemblies in soft matter.

## Introduction

Collective orientation plays an important role in a large variety of self-assembly processes, such as wound healing in biology and topological evolution in soft matter. However, non-destructive optical characterization of three-dimensional (3D) orientation in complex assemblies remains a significant challenge. To access the 3D orientation, probes with anisotropic shape and strongly anisotropic optical properties can be exploited. Depending on the origin of the optical anisotropy, various techniques exist to retrieve the 3D orientation of single particles, such as fluorescence polarization microscopy^1–4^, point spread function (PSF) engineering^5,6^, and defocused imaging^7,8^. However, techniques that require the individual probes to be optically resolved cannot be applied for characterizing dense assemblies. Moreover, widefield microscopy approaches lack optical sectioning, preventing tomographic imaging of orientation in thick assemblies.

Due to these challenges, there are only limited demonstrations in ensemble systems. The most developed technique for characterizing ensembles is polarization resolved fluorescence microscopy and has been demonstrated both for linear fluorescence and two-photon fluorescence using molecular dyes in biological systems^9,10^. This technique measures the intensity fluctuations when rotating the polarization axis of the excitation to reconstruct the orientation of the emission dipole. As it measures global intensity, it is sensitive to noise and requires measurements at many different analyzer angles to minimize the effect of background intensity fluctuations. Label-free techniques based on IR spectroscopy^11^, non-linear optical processes^12–15^, birefringence^16^ or the dielectric tensor^17^ exist, but require complex optical setups and are limited to systems with suitable inherent optical properties.

Lanthanide-doped nanoparticles are a promising, yet often overlooked, candidate for orientation sensing due to their strongly polarized photoluminescence. In most cases, this photoluminescence originates from energy transitions within the sublevels of the 4 f orbitals of the lanthanide ion, which are influenced by the crystal-field of the host matrix^18^. Anisotropy of the crystal structure results in polarized emission, seen as a change in the spectral line shape when rotating an analyzer with respect to the crystal axis. Unlike other optical probes providing a single dipolar component, lanthanide’s polarized luminescence is linked to multiple magnetic (MD) and electric (ED) dipoles. A single spectroscopic measurement of the polarized emission thus allows to simultaneously observe multiple dipoles manifesting distinct orientations (governed by the crystal field) and natures of polarization (according to MD and ED)^1^. This makes it possible to spectroscopically resolve the 3D orientation of a single crystal, without the need for multiple observation angles. In practice, this can be done by a spectroscopic analysis of the polarized emission of an unknown system with respect to the reference spectra obtained from an ideal assembly with a controlled orientation.

The relatively large size and aspect ratio of most lanthanide-doped nanorods, as compared to molecular probes, make them excellent candidates for the study of collective orientation either through self-assembly or under the influence of external stimuli, leading to their orientation. At sufficiently high concentrations, suspensions of such anisotropic nanoparticles exhibit liquid crystal (LC) behavior and can spontaneously form intricate self-assemblies^19,20^. Such intrinsically labeled systems form a powerful platform for the study of self-assembly mechanics and the energy balance in soft matter. Moreover, nanoparticles with a large aspect ratio can easily be aligned by external forces, such as electric fields and hydrodynamic shear. Indeed, measurements of the polarized emission of lanthanide-doped nanoparticles have been demonstrated to probe the local viscosity and shear stress in fluids^21–24^.

Although determining the 3D orientation of a single emitter is relatively simple, tomographic mapping of orientation in dense ensembles remains an open challenge. For ensembles, the 3D orientation is not only described by the average orientation, the director $\vec{{{{\bf{n}}}}}$, but also by the degree of collective order known as the order parameter S. However, this additional degree of freedom makes it impossible to get a unique solution for S and the out-of-plane tilt angle, as both a loss in order and an out-of-plane tilt affect the perceived optical anisotropy in similar ways. This effect is not limited to lanthanide luminescence but plagues all orientation analysis methods based on a single viewing angle. In practice, orientation tomography is therefore performed either by assuming perfect order within the measurement volume^16,17^, or by assuming $\vec{{{{\bf{n}}}}}$ being completely parallel or perpendicular to an interface or specified plane based on prior knowledge of the system^9,10,25^. To extend our technique to ensembles with more complex configurations (intermediate values of order and tilt), an advanced theoretical framework needs to be established. The additional degree of freedom also emphasizes the need for high-quality reference data, as spectra collected from a suboptimal sample could lead to significant errors in the orientation analysis. For ensembles with unknown orientation, it is thus of high importance that the quality of the reference sample is verified.

To achieve high-resolution imaging of lanthanide luminescence for 3D orientation tomography, several practical challenges need to be addressed. First, the luminescence lifetime of lanthanides is relatively long, up to 10^−3^ s, which is incompatible with the fast beam scanning mechanisms of conventional confocal microscope systems employing galvano-mirrors, which typically have a minimum scan rate of 10^−6 ^s per pixel^18^. Secondly, the large Stokes shift (Δλ~200-300nm), i.e., the difference in excitation and emission wavelengths, can deteriorate the axial resolution due to chromatic aberrations induced by the optics, resulting in a mismatch of focal planes. Most commercially available aberration-corrected achromatic microscope objectives are not capable of correcting such large Stokes shifts of lanthanide luminescence. Finally, as the orientation measurements require polarization-resolved spectroscopy, special care should be taken when designing the optical configuration to minimize distortions in the polarization state through the optical path.

Here, we expand on our previous work by providing a complete methodology for spectroscopic orientation analysis in ensembles with an unknown 3D orientation. We first provide some background on lanthanide luminescence and provide a method to obtain high-quality reference spectra. To make the technique accessible to implement, we provide a thorough description of the confocal microscopy setup adapted to imaging lanthanides, achieving a spatial resolution of 0.5 μm (lateral)-by-1 μm (axial), and highlight several practical concerns. The theoretical framework required for orientation analysis of ensembles with arbitrary orientation is then developed. Finally, the technique is demonstrated in both a dense LC assembly and a dilute, flowing suspension of nanoparticles in a microfluidic channel.

## Results

### Polarized luminescence and obtaining reference spectra

**Fig. 1:**
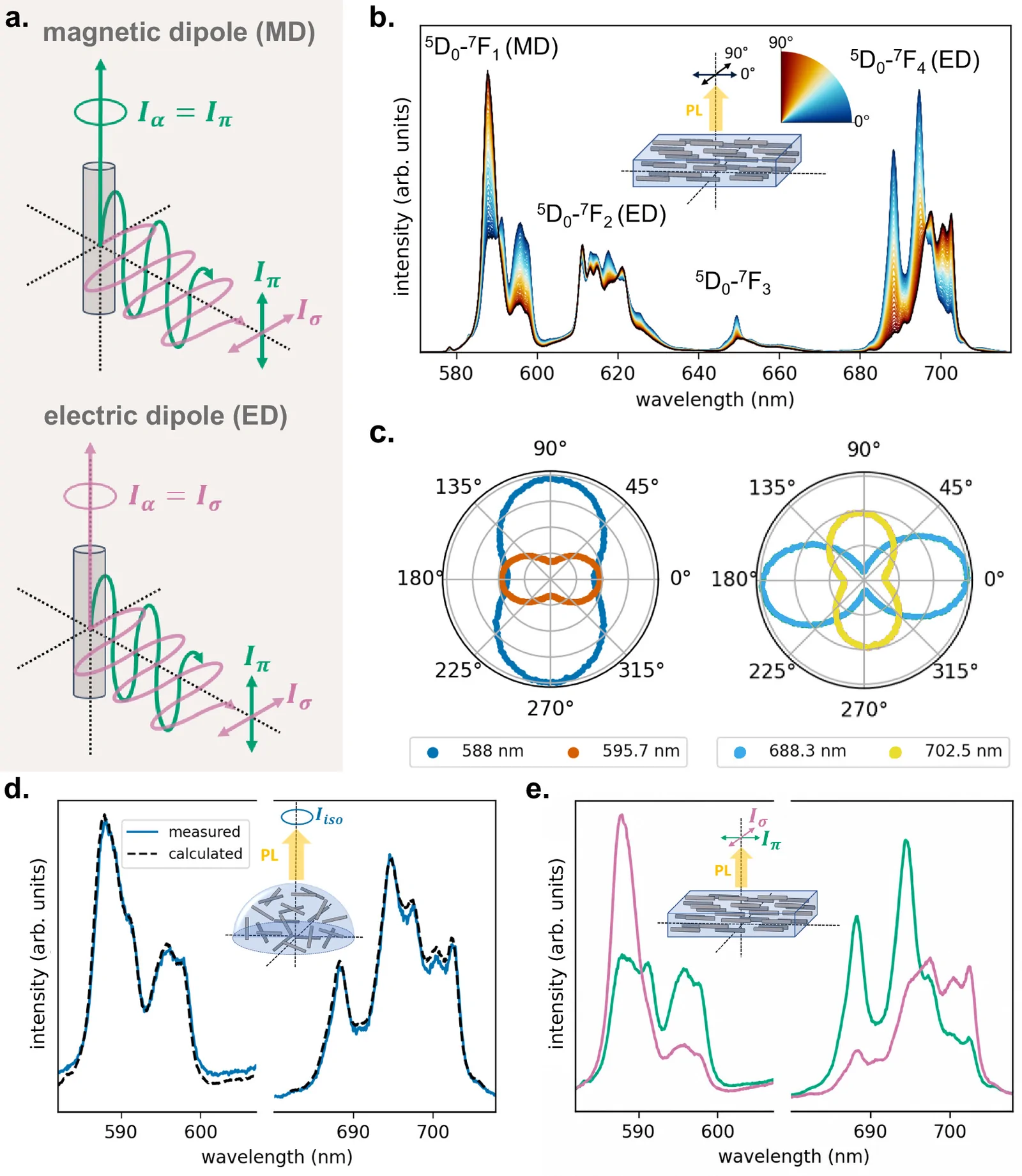
Polarized emission of hexagonal LaPO_4_:Eu nanorods. **a** Schematic illustration of the polarized emission components for magnetic dipole (MD) and electric dipole (ED) transitions. **b** Polarized emission spectra measured in a dense, planar liquid crystal (LC) assembly when rotating the analyzer from 0° to 90° with respect to the horizontal alignment axis. **c** Intensity for a full rotation of the analyzer of the most strongly polarized emission peaks in the ^5^D_0_-^7^F_1_ and ^5^D_0_-^7^F_4_ transitions, respectively. **d** Reference ${I}_{\sigma }$ and ${I}_{\pi }$ spectra obtained in a highly aligned, planar LC sample. **e** Comparison of the measured isotropic emission in a diluted suspension and the isotropic spectrum calculated based on the reference ${I}_{\sigma }$ and ${I}_{\pi }$ from d) using Eqs. 4–5.

The key to using lanthanides for orientation measurements is understanding the nature of their polarized emission originating from two different types of dipoles (MD and ED). We have previously performed in-depth studies on the polarized emission of both LaPO_4_:Eu^3+^ and NaYF_4_:Eu^3+^ nanocrystals and will briefly discuss the main aspects here^1,26,27^. The polarized emission of a single nanorod can be decomposed into three components: the unpolarized axial emission ${I}_{\alpha }$, and the radial emission polarized parallel and perpendicular to the rod axis ${I}_{\pi }$ and ${I}_{\sigma }$ respectively. Moreover, the axial emission couples with either the π or σ component depending on whether the transition is associated to a MD or ED (Fig. 1a), such that

1 $${I}_{\alpha,{{{\rm{ED}}}}}\left(\lambda \right)={I}_{\sigma,{{\rm{ED}}}}\left(\lambda \right)$$

2 $${I}_{\alpha,{{{\rm{MD}}}}}\left(\lambda \right)={I}_{\pi,{{\rm{MD}}}}(\lambda )$$

The full characterization of the PL can therefore be achieved by measurement of the radial emission spectra ${I}_{\pi }$ and ${I}_{\sigma }$ and applying Eqs. 1–2. Moreover, we have previously shown that the fine line spectra obtained from aligned assemblies are identical to those of isolated nanocrystals^1,22,26^. Instead of relying on the weak emission of a single particle, it is therefore advantageous to use highly aligned planar assemblies to obtain the ${I}_{\pi }$ and ${I}_{\sigma }$ spectra. Figure 1b shows the emission collected from a planar nematic liquid crystal of hexagonal LaPO_4_:Eu nanorods for varying analyzer angles. Each group of peaks corresponds to a specific energy transition as indicated in Fig. 1b. The ^5^D_0_-^7^F_1_ transition (585-600 nm) originates from a MD, whereas the ^5^D_0_-^7^F_2_ (608-625 nm) and ^5^D_0_-^7^F_4_ (680-705 nm) transitions are associated to EDs. The narrow peaks within each transition are linked to the crystal field around the Eu^3+^ ion and the precise line spectra can therefore vary greatly between different Eu^3+^ hosts, as seen when comparing the emission spectra of the hexagonal (Fig. 1b) and monoclinic (Fig. S7) LaPO_4_:Eu phases. Each subpeak of the radial emission follows the dipolar model such that

3 $${I}_{{{{\rm{rad}}}}}\left(\chi \right)={I}_{\pi }\cdot {\cos }^{2}\chi+{I}_{\sigma }\cdot {\sin }^{2}\chi$$

where χ is the angle between the analyzer and the c-axis of the crystal lattice. Subpeaks are either dominated by the ${I}_{\pi }$ or ${I}_{\sigma }$ contribution, resulting in orthogonal dumbbells seen in the polar diagram (Fig. 1c). The narrowness of the dumbbells represents the degree of polarization (DOP = $\left({I}_{\pi }-{I}_{\sigma }\right)/\left({I}_{\pi }+{I}_{\sigma }\right)$). For the hexagonal LaPO_4_:Eu nanocrystals, the most polarized peaks are around 588 nm and 596 nm for MD and 688 nm and 703 nm for ED. For orientation probing applications, it is best to use the peaks with the highest DOP, and we therefore use the ^5^D_0_-^7^F_4_ transition as the considered ED transition.

The reference ${I}_{\pi }$ and ${I}_{\sigma }$ spectra can be collected from the aligned sample by measuring the luminescence with the analyzer parallel and perpendicular to the orientation axis respectively (Fig. 1d). It is important to measure the reference spectra with the same optical setup as the orientation measurements, as the fine line shape is affected by parameters such as the numerical aperture of the objective and the slit size of the spectrometer. The accuracy of the spectroscopic orientation analysis relies on the quality of these measured reference ${I}_{\pi }$ and ${I}_{\sigma }$ spectra. To avoid collecting the axial emission, the assembly should be oriented parallel to the observation plane and be as close to perfect alignment as possible. However, it is challenging to experimentally evaluate the 3D alignment quality of samples, and thus the quality of the measured reference spectra, by non-destructive optical methods. Instead, we propose to evaluate the quality of in-plane alignment of the reference sample by using the collected reference spectra to simulate the isotropic spectrum and comparing it to the experimentally measured isotropic spectrum. For a collection of completely disordered particles, the emission measured for all analyzer angles will be an equal contribution of ${I}_{\pi }$, ${I}_{\sigma }$ and ${I}_{\alpha }$. Based on the relationship expressed in Eqs. 1–2, the isotropic emission ${I}_{{{{\rm{iso}}}}}$ thus follows

4 $${I}_{{{{\rm{iso}}}},{{\rm{ED}}}}\left(\lambda \right)=\frac{1}{3}\left({I}_{\pi,{{\rm{ED}}}}\left(\lambda \right)+2\cdot {I}_{\sigma,{{\rm{ED}}}}\left(\lambda \right)\right)$$

5 $${I}_{{{\rm{iso}}},{{\rm{MD}}}}\left(\lambda \right)=\frac{1}{3}\left(2\cdot {I}_{\pi,{{\rm{MD}}}}\left(\lambda \right)+{I}_{\sigma,{{\rm{MD}}}}\left(\lambda \right)\right)$$

To directly measure ${I}_{{{\rm{iso}}}}$, a droplet of diluted nanorod suspension ( < 0.1 vol%), corresponding to the system to be studied, is placed on a coverslip and its emission spectrum is recorded for a full rotation of the analyzer to verify the lack of collective order. This measured spectrum is then compared to the simulated isotropic spectrum based on the reference ${I}_{\pi }$ and ${I}_{\sigma }$ using Eqs. 4–5 (Fig. 1e). We indeed find a strong agreement between the experimental and simulated spectra, thus validating the quality of the reference spectra taken from a nematic assembly. This indicates that the sample is highly planar, as an out-of-plane tilt of the director would distort the calculated isotropic spectrum (Fig. S8). In practice, we acquire multiple measurements in the reference sample at different locations and finally select the reference spectra that have both a high DOP and a good fit between the experimental and simulated isotropic spectrum.

### Polarization resolved microscopy for lanthanide luminescence

**Fig. 2:**
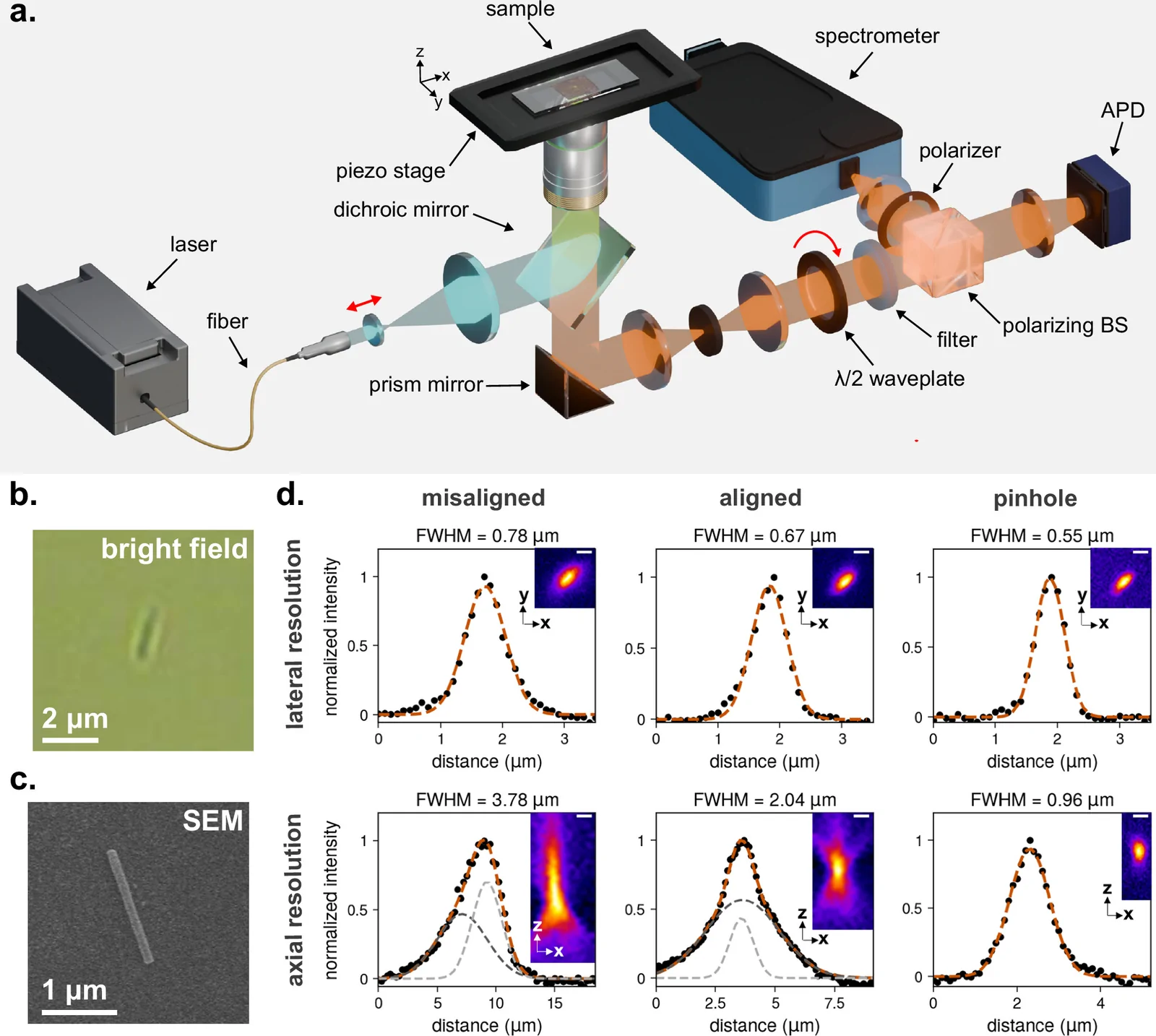
Optical setup and resolution. **a** Experimental confocal micro-spectroscopy setup. A continuous fiber-coupled laser (λ_exc_ = 394 nm) is expanded using two doublets (f_1_ = 50 mm, f_2_ = 100 mm) to fit the back pupil of the objective (60x oil immersion, NA = 1.49). The first doublet is mounted on a z-axis stage to control the focus of the excitation beam. Point scanning is performed using a three-axis piezoelectric stage. The collected emission is focused on a 50 μm pinhole using a tube lens (f = 200 mm) and collimated with a doublet (f = 150 mm). The polarization state is selected by a λ/2 waveplate on a motorized rotating mount, followed by a polarizing beam splitter. The s-polarized light is reflected to a spectrometer through a linear polarizer, and the p-polarized light is transmitted to an avalanche photodiode (APD). **b, c** Bright-field and scanning electron microscopy (SEM) images of a NaYF_4_:Eu^3+^ nanorod used for the alignment and resolution measurements. **d** Spatial resolution measurements using a single NaYF_4_:Eu^3+^ nanorod for various stages in the alignment procedure. Insets show the constructed APD intensity image with scale bars of 1 μm. The resolution is approximated as the full-width half-maximum (FWHM) of the intensity profile plotted across the diameter of the nanorods. Measurements (black dots) were fit with either a single Gaussian fit (orange dashed lines) of as the sum of two Gaussians (gray dashed lines).

We will first give a general overview of the microscopy setup, followed by some practical recommendations regarding optimizing spatial resolution and minimizing polarization distortions. To achieve polarization-resolved tomographic imaging, a commercial inverted microscope (Nikon Eclipse Ti) is adapted, as illustrated in Fig. 2a. Photoluminescence is excited by a monochromatic laser (λ_exc_ = 394 nm) corresponding to the narrow absorption peak of the ^7^F_0_-^5^L_6_ transition of the Eu^3+^ ions^22^. The collected emission (λ_em_ ~ 580 - 710 nm) is focused onto a pinhole to obtain optical sectioning. The polarization state is selected using an achromatic λ/2 waveplate on a motorized rotating mount, followed by a polarizing beam splitter. The s-polarization is reflected to a spectrometer equipped with a CCD camera and the p-polarization is transmitted to an avalanche photo diode (APD). A linear polarizer is placed between the beam splitter and the spectrometer to remove any remaining p-polarized light. To account for the long lifetimes of lanthanides, imaging is achieved by point scanning using a piezoelectric stage without constraints on the dwelling time at a given position.

The spatial resolution of the setup is predominantly determined by the numerical aperture (NA) of the objective, as the theoretical resolution of a confocal microscope scales inversely with NA^28,29^. We therefore work with a high NA objective (NA = 1.49) and high refractive index immersion oil (n = 1.51). Based on the equations reported in the literature^29^, the spatial resolution should roughly be 0.14 μm by 0.3 μm (for λ_exc_ = 394 nm). For the experimental measurements of the resolution, we use NaYF_4_:Eu nanoparticles, which are long enough to easily be identified, but whose width (117  ±  24 nm) is on the order of the diffraction limit (Fig. 2b, c)^1^. In principle, different sub-resolution luminescent probes could be used as long as their excitation and emission wavelengths match those of the target lanthanides. The resolution can then be extracted from images of the total intensity recorded with the APD, considering the full width half maximum (FWHM) of the intensity profile across the diameter of the particle. Fig. 2d shows typical images (insets) and intensity profiles for both the lateral and axial direction throughout different stages of the alignment process. The first column represents the performance of a commercial microscope, where the excitation laser would be perfectly collimated, which doesn’t address the mismatch of the excitation focal plane with the emission focal plane. The middle column shows the well-aligned setup without a pinhole, and the right column shows the setup with a pinhole in the emission path.

We find a slight improvement in lateral resolution, with an optimal resolution of 0.55 μm, but the biggest impact is on axial resolution. This can be attributed to significant chromatic aberration caused by the large difference in excitation and emission wavelength. This is clearly observed by the two Gaussian contributions to the intensity profile in Fig. 2d (left column). To correct this, the excitation beam was adjusted to be slightly converging, which we control by fine tuning the z-position of the first plano convex lens in the telescope setup, ideally without any pinhole (Fig. 2a, red arrow). The resulting intensity profile consists of two Gaussians with the same center, and we typically find a FWHM of 2 μm. Introducing the 50 μm pinhole in the emission path further enhances the axial resolution to roughly 1 μm. Conventionally, the optimal pinhole size is expected to be around the size of the diameter of the Airy disk (${D}_{{{\rm{Airy}}}}$ ~ 32 μm for λ ~ 650 nm and 60x magnification)^30^. However, picking a slightly larger pinhole gives similar performance without losing too much intensity.

As the orientation analysis is based on polarization-resolved spectroscopy, it is important to avoid any distortions to the polarization state throughout the imaging path. This can be verified by measuring the emission from both aligned and isotropic ensembles for different analyzer angles. For aligned samples the subpeaks should follow a dipolar model (Fig. 1c). However, the initial optical configuration, produced butterfly-shaped plots of $I\left({{{\rm{\chi }}}}\right)$ (Fig. S9). This artefact seems to have originated from the use of a conventional metal mirror whose reflectance is somewhat polarization dependent likely due to its coating, as the expected dipolar model was retrieved when changing to an uncoated prism mirror. This illustrates the importance of carefully considering each element in the optical path and its influence to the polarization. Similarly, it is preferential for the emission beam to be transmitted through any dichroic beam splitters, rather than reflected. Finally, as the spectrometer grating is also sensitive to polarization, the linear-polarization axis should be fixed with a linear polarizer (or polarizing beam splitter). The effective analyzer angle should thus be selected by rotating a λ/2 waveplate in front of the fixed linear polarizer. It should be noted that the technique is not sensitive to the polarization of the excitation beam, as for lanthanides the polarization of the emission is decoupled from the excitation^18^. This is because the excitation and emission involve different energy transitions, ^7^F_0_-^5^L_6_ and ^5^D_0_-^7^F respectively, separated by a non-radiative relaxation from ^5^L_6_ to ^5^D_0_^31^.

### Orientation analysis of arbitrary ensembles

**Fig. 3:**
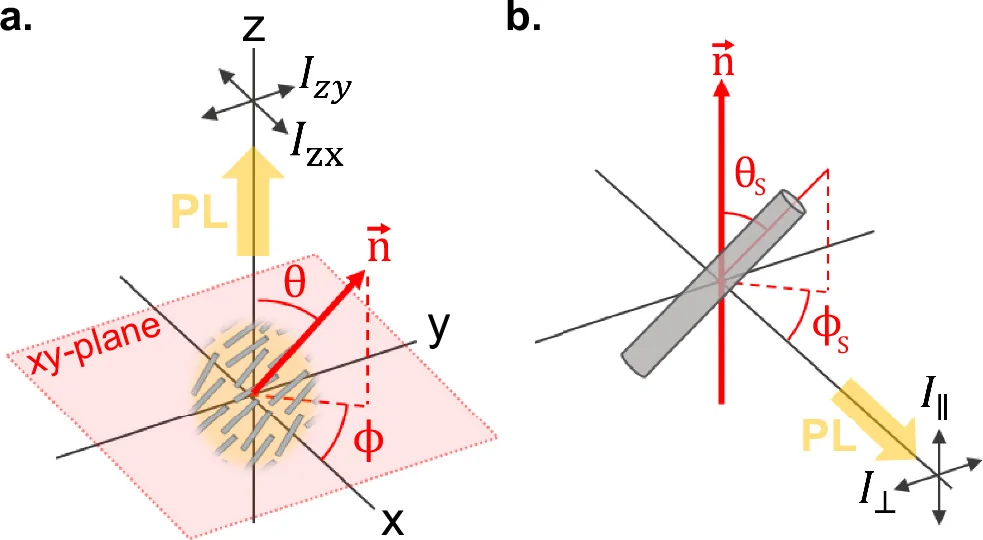
Schematic illustration of the coordinate systems. **a** The average orientation, director $\vec{{{{\bf{n}}}}}(\theta,\phi )$ in the laboratory frame with respect to direction of the photoluminescence (PL) collection. **b** The orientation of a single particle within the ensemble with respect to $\vec{{{{\bf{n}}}}}$.

For an ensemble of nanoparticles, its collective orientation is quantified by the average orientation known as the director $\vec{{{{\bf{n}}}}}\left(\theta,\phi \right)$, where θ and ϕ are the polar and azimuthal angles shown in Fig. 3a, and by the order parameter S which describes the local degree of order (fully disordered S = 0, perfect collective alignment S=1). S is expressed by the angle between individual particles and $\vec{{{{\bf{n}}}}}$, denoted by ${\theta }_{s}$ (Fig. 3b), such that

6 $$S=\frac{3\cdot \left\langle {\cos }^{2}{\theta }_{s}\right\rangle -1}{2}$$

where $\langle {\cos }^{2}{\theta }_{s}\rangle$ describes the average over all rods in the focal volume. The emission spectra measured with the analyzer parallel to the x-axis ${I}_{{zx}}$ or to the y-axis ${I}_{{zy}}$ can then be expressed in terms of the reference (${I}_{\pi }$ and ${I}_{\sigma }$) and the collective orientation ($\vec{{{{\bf{n}}}}}\left(\theta,\phi \right)$ and S), according to the detailed derivation in the SI.

For MD transitions

7 $${I}_{{zx},{{\rm{MD}}}}\left(\lambda \right)=	 {I}_{\pi,{{\rm{MD}}}}\left(\lambda \right)\cdot \left(\frac{2}{3}+S\cdot \left(\frac{1}{3}-{\sin }^{2}\theta \cdot \left(1-{\cos }^{2}\phi \right)\right)\right) \\ 	+{I}_{\sigma,{{\rm{MD}}}}\left(\lambda \right)\cdot \left(\frac{1}{3}-S\cdot \left(\frac{1}{3}-{\sin }^{2}\theta \cdot \left(1-{\cos }^{2}\phi \right)\right)\right)$$

8 $${I}_{{zy},{{\rm{MD}}}}\left(\lambda \right)=	 {I}_{\pi,{{\rm{MD}}}}\left(\lambda \right)\cdot \left(\frac{2}{3}+S\cdot \left(\frac{1}{3}-{\sin }^{2}\theta \cdot {\cos }^{2}\phi \right)\right) \\ 	+{I}_{\sigma,{{\rm{MD}}}}\left(\lambda \right)\cdot \left(\frac{1}{3}-S\cdot \left(\frac{1}{3}-{\sin }^{2}\theta \cdot {\cos }^{2}\phi \right)\right)$$

And similarly, for ED transitions

9 $${I}_{{zx},{{\rm{ED}}}}\left(\lambda \right)=	 {I}_{\pi,{{\rm{ED}}}}\left(\lambda \right)\cdot \left(\frac{1}{3}-S\cdot \left(\frac{1}{3}-{\sin }^{2}\theta \cdot {\cos }^{2}\phi \right)\right) \\ 	+{I}_{\sigma,{{\rm{ED}}}}\left(\lambda \right)\cdot \left(\frac{2}{3}+S\cdot \left(\frac{1}{3}-{\sin }^{2}\theta \cdot {\cos }^{2}\phi \right)\right)$$

10 $${I}_{{zy},{{\rm{ED}}}}\left(\lambda \right)=	 {I}_{\pi,{{\rm{ED}}}}\left(\lambda \right)\cdot \left(\frac{1}{3}-S\cdot \left(\frac{1}{3}-{\sin }^{2}\theta \cdot \left(1-{\cos }^{2}\phi \right)\right)\right) \\ 	+{I}_{\sigma,{{\rm{ED}}}}\left(\lambda \right)\cdot \left(\frac{2}{3}+S\cdot \left(\frac{1}{3}-{\sin }^{2}\theta \cdot \left(1-{\cos }^{2}\phi \right)\right)\right)$$

Furthermore, for both MD and ED transitions the emission measured with the analyzer at 45° with respect to the x-axis ${I}_{z45^\circ }$ can be expressed in terms of ${I}_{{zx}}$ and ${I}_{{zy}}$, such that

11 $$2{I}_{z45^\circ }\left(\lambda \right)={I}_{{zx}}\left(\lambda \right)+{I}_{{zy}}\left(\lambda \right)+\left({I}_{{zx}}\left(\lambda \right)-{I}_{{zy}}\left(\lambda \right)\right)\cdot \tan \left(2\phi \right)$$

Whereas ϕ can be determined directly from Eq. 11 or combinations of Eqs. 7–10, there are no unique solutions for S and ${\sin }^{2}\theta$. Within this framework, some assumptions need to be made to resolve the system. Here two practical examples will be discussed where either S or θ is known or can be reasonably estimated. As was previously demonstrated in the case of single particles^1^, the orientation can be extracted from these expressions and the experimental measurements either by a spectral curve fitting or by a ratiometric approach. In the spectral fitting approach, the measured emission spectra are fit to the corresponding expressions using an optimization algorithm. In the second approach, the expressions are redefined in terms of the ratio between the area under the curve (AUC) of two subpeaks from the same transition. This allows the analysis to be performed using single scalar values, rather than considering the fine line spectra. In the next section, examples of both approaches will be given and additional information can be found in the supplementary information.

### Orientation analysis for highly ordered assemblies

**Fig. 4:**
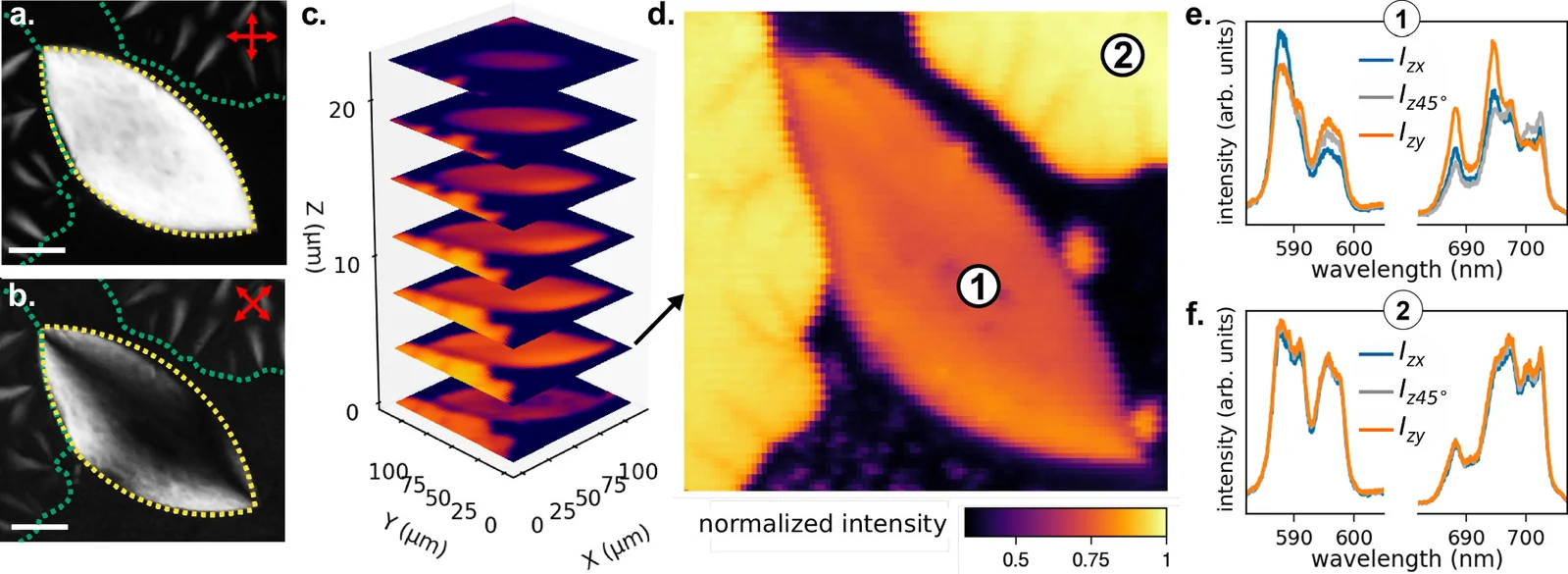
Highly ordered assembly in a liquid crystal (LC) of LaPO_4_:Eu nanorods in ethylene glycol. **a, b** Polarized optical microscopy (POM) images of a tactoids (outlined in yellow) surrounded by flower domains (outlined in green) taken at different orientations of crossed polarizers as indicated by the red arrows. The scale bars represent 25 μm. These images represent the same birefringence textures of many tactoids and flower domains constantly observed on more than five samples prepared independently. **c** Tomography of photoluminescence (PL) intensity. **d** Normalized intensity for the xy-plane indicated by the black arrow. **e, f** Emission spectra for three different analyzer angles taken at the positions indicated in d.

First, we consider a highly ordered nematic LC assembly of LaPO_4_:Eu^3+^ nanorods, in which *S* is approximated to be constant throughout the volume. Such colloidal LCs can form a variety of 3D structures, ranging from largely planar to strongly vertical configurations^20^. However, the conventional polarized optical microscopy (POM) can only resolve 2D projections of the 3D structures. As a proof of concept, we utilize a sample which contains two kinds of domains; a nematic tactoid and a flower-like structure indicated by the yellow and green dashed lines in the POM images respectively (Fig. 4a, b). Comparing POM images taken with two different orientations of the crossed polarizers gives qualitative information on the director field (see methods). Based on the POM images, the tactoid is largely planar whereas the flower domain is likely close to vertical, making this an excellent sample to evaluate the orientation tomography technique (Fig. 4a, b).

Tomographic measurements of the PL are performed (Fig. 4c) such that for each voxel three spectra are collected with the analyzer parallel to the x- and y-axes and one in between at 45° respectively. The dense, highly ordered nematic domains can be identified from the high PL intensity compared to the surrounding, low density isotropic phase, and the 3D reconstruction (Fig. S10) shows that the tactoids has a flattened rugby-ball shape and the flower domain consists of sharp ridges protruding from the substrate. As expected from POM, PL spectra taken within the tactoid are strongly polarized (Fig. 4e), indicating a planar alignment, whereas the spectra collected from the flower domain are unpolarized and closely match ${I}_{\alpha }$ (Fig. 4f), indicating vertical alignment.

**Fig. 5:**
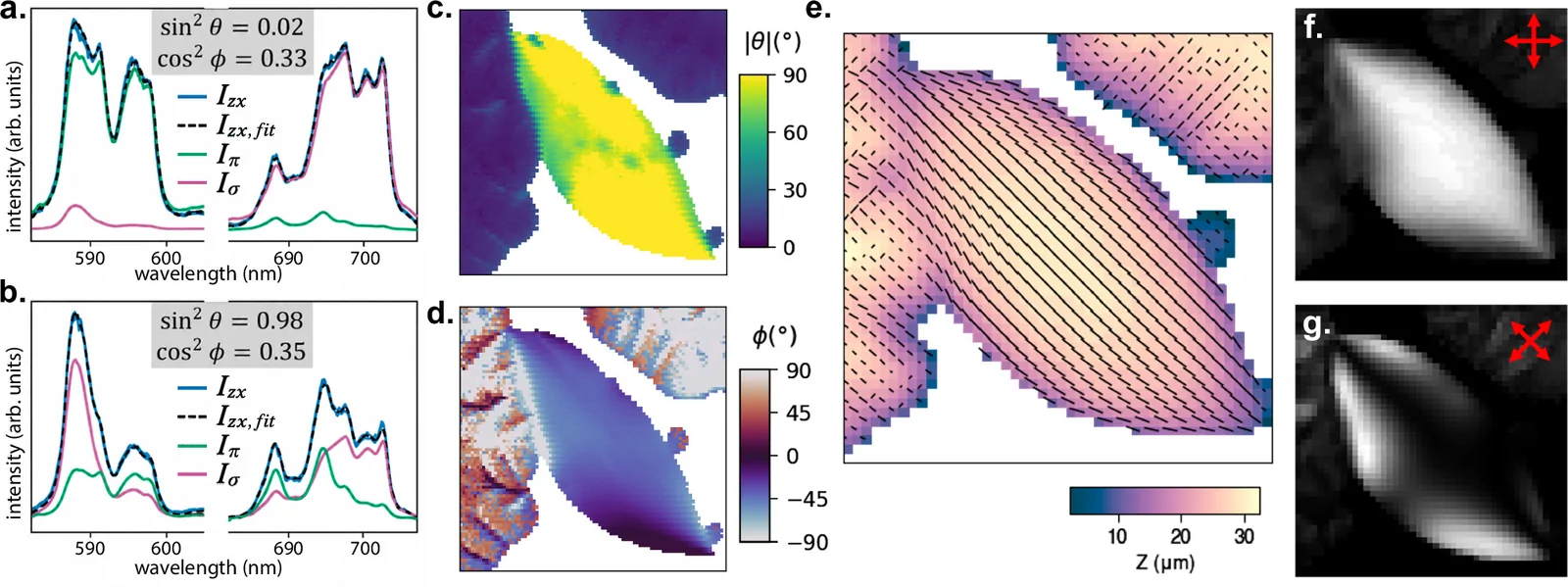
Spectral fitting analysis or highly ordered assembly. **a, b** Spectral fitting of ${I}_{{zx}}$ for position 1 and 2 from Fig. 4e, f respectively. The resulting fit is shown as black dashed line consisting of a weighted sum of the reference ${I}_{\sigma }$ and ${I}_{\pi }$. **c, d** Obtained values of $\left|\theta \right|$ and ϕ respectively for the xy-plane shown in Fig. 4d. **e** Projection of $\vec{{{{\bf{n}}}}}$ on the top interface of the ordered nematic volume. **f, g** Simulated birefringence patterns based on the calculated $\vec{{{{\bf{n}}}}}$ throughout the volume for two orientations of crossed polarizers (red arrows), equivalent to the polarized optical microscopy (POM) images in Fig. 4a, b.

To resolve the local 3D orientation for each voxel of the measurement, an approximation of the global value of *S* needs to be made. Here, we consider that within the flower domain the orientation close to the substrate is vertical due to strong anchoring between the LC and the substrate, and use our previously reported method to find S = 0.85 (Fig. S4)^32^. Next, to find the values of ${\sin }^{2}\theta$ and ${\cos }^{2}\phi$, at least two different expressions from Eqs. 7–10 are required, with one depending on ${\sin }^{2}\theta \cdot \left(1-{\cos }^{2}\phi \right)$ and the other on ${\sin }^{2}\theta \cdot {\cos }^{2}\phi$. Here, we consider ${I}_{{zx}}$ for both MD and ED transitions and first apply the spectral fitting approach based on Eqs. 7 and 9. Figure 5a, b shows examples of the spectral fit (black dashed lines) of the two ${I}_{{zx}}$ spectra (blue) as the weighted sum of the ${I}_{\pi }$ and ${I}_{\sigma }$ spectra (green and pink lines). This spectral fitting approach is then repeated for all voxels, yielding the ${\sin }^{2}\theta$ and ${\cos }^{2}\phi$, values (Fig. S11) which give the absolute orientation $\left|\theta \right|$ and $\left|\phi \right|$ (Fig. 5c, d). As expected, we find that the rods are oriented vertically in the flower domains ($\left|\theta \right|\approx 0^\circ$) and are close to parallel within the tactoids itself ($\left|\theta \right|\approx 90^\circ$) (Fig. 5c). Although the spectral fit directly yields the absolute orientation $\left|\theta \right|$ and $\left|\phi \right|$, additional considerations are needed to eliminate their mirror angles. Due to the single viewing angle with respect to the z-axis, it is not possible to distinguish $\pm \theta$ spectroscopically, instead we rely on the boundary condition that the director should be parallel to the I-N interface (Fig. S12). To eliminate the mirror angle of ϕ, we consider ${I}_{z45^\circ }$ as described in the SI (Fig. 5d). Figure 5e displays $\vec{{{{\bf{n}}}}}$ as projected on the top I-N interface, where the length of the bars represents the projection on the xy-plane. $\vec{{{{\bf{n}}}}}$ follows the contours of the tactoids, which is consistent with previous observations^33^.

Finally, to validate the accuracy of the obtained orientation profile, we create simulated POM images (as described in the SI) and compare them to the measured images. Indeed, simulated profiles Fig. 5f, g show an excellent agreement with the images in Fig. 4a, b.

It should be noted that for thick, strongly birefringent samples, the polarization state could be rotated as the PL propagates through the sample. In the system discussed here, this impact is negligible as the director field is largely homogenous throughout the depth of the LC. However, in the case of an assembly with a strongly varying in-plane orientation, such as chiral LCs, the birefringence could cause a significant distortion in the measured orientation. A more detailed discussion and a correction protocol regarding this issue can be found in the SI.

Other combinations of Eqs. 7–11 can also be used to retrieve the orientation and all give comparable results. For this demonstration the ${I}_{{zy}}$ spectra were not needed for the orientation analysis as both MD and ED transitions were considered on the ${I}_{{zx}}$ spectra. The total dataset could therefore be reduced by one third, which would in turn decrease the total scanning time required. However, when working with a spectrometer grating with a smaller spread, it could be advantageous to consider only the highly polarized ^5^D_0_-^7^F_4_ transition and perform the analysis on only this ED transition. Finally, the orientation could also be reconstructed based on the ratiometric approach as detailed in the SI.

Building on the method demonstrate here, an advanced analysis of a LC morphogenesis phenomenon has been performed and presented in a separate report^20^.

### Orientation analysis for ensembles with unknown order parameter

As a second example system, we consider a flowing suspension of nanoparticles in microfluidic channels. In such systems, accurate measurements of the order parameter, rather than the 3D orientation, are crucial, especially for the application of shearmetry. We have previously shown that the direct correlation between S and the shear stress exerted on suspensions of nanorods can be exploited to characterize fluid flows in situ^22–24^. To get unique solutions for the orientation equations, we previously approximated that the director was fully horizontal ($\theta=90^\circ$) throughout the whole depth of the system. Moreover, in our comparative study on the influence of particle size, we used a fixed director ($\phi=0^\circ,\theta=90^\circ$) to generate the maps of S^24^. Evidently, such approximations can induce inaccuracies in the results, which will be addressed in the following section.

As previously stated, the tilt angle $\theta \,$ and S cannot be determined independently. Measuring S thus require an estimation on θ. Here we will first discuss the case where $\theta \approx$ 90° (planar alignment) at the center height of a symmetric channel, followed by an approach for mapping S in vertical cross sections by estimating the profile of θ. Although the demonstration here is limited to the determination of the collective orientation itself, the calculated values of S can be easily converted to shear maps by applying a calibration curve (Fig. S13).

In the case where θ = 90°, Eqs. 7–10 can be simplified as all ${\sin }^{2}\theta$ terms become 1. However, under this condition, expressions 7 and 10 contain the term $S\cdot \left(\frac{1}{3}-{\cos }^{2}\phi \right)$, which goes to 0 when ${\cos }^{2}\phi=\frac{1}{3}$ and the resulting spectra are no longer a function of S. Similarly, Eqs. 8 and 9 lose the dependency of S for ${\cos }^{2}\phi=\frac{2}{3}$. This means there are some orientations of the director for which the measured spectrum doesn’t depend on S, it will always appear isotropic. To minimize the impact of these “blind angles”, it is important to include the ${I}_{z45^\circ }$ spectra in the analysis. In the case of the AUC ratiometry, this is done by calculating ϕ first using Eq. 11 (which considers all spectra) and using it as input to solve for S. For the spectral fitting approach, the best results are found when fitting ${I}_{z45^\circ }$ and ${I}_{{zx}}$ (and/or ${I}_{{zy}}$) simultaneously.

**Fig. 6:**
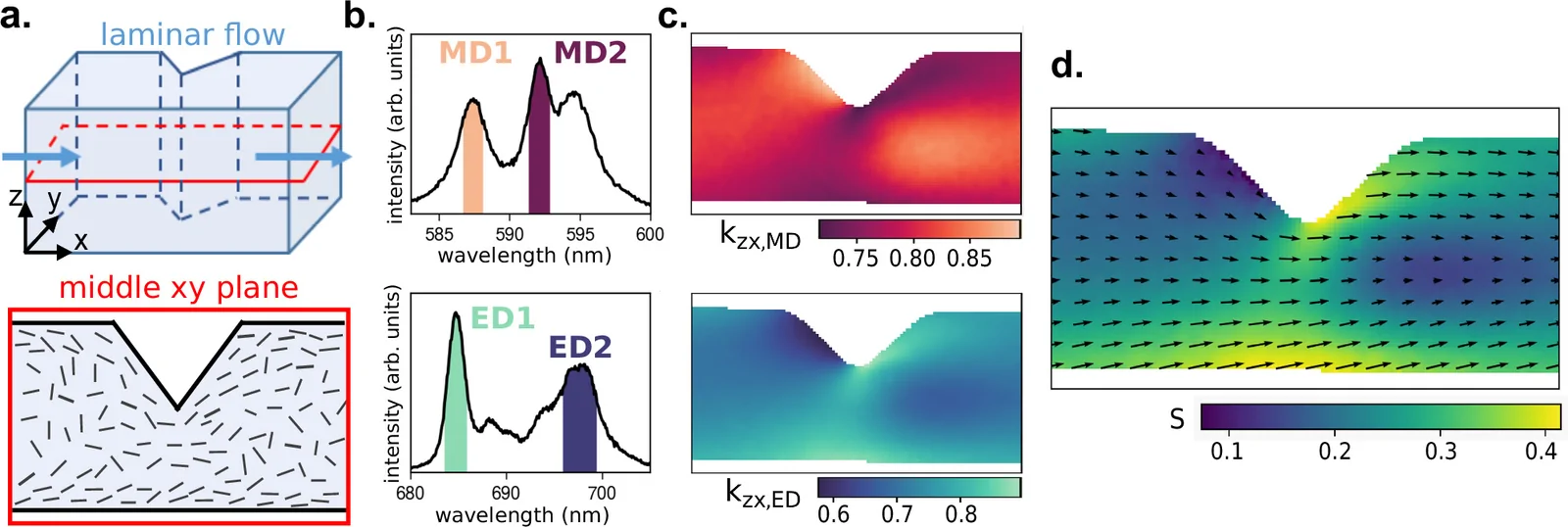
Planar orientation at the middle height of a microfluidic channel. **a** Schematic illustration of the microfluidic channel with a triangular barrier and a 50-by-50 μm^2^ cross section. A dilute suspension of monazite LaPO_4_:Eu nanorods flows through the channel at 10 nL/min, a flow rate which corresponds to the high sensitivity region in the calibration curve (Fig. S13). Measurements of the polarized photoluminescence (PL) are taken at the middle plane where $\vec{{{{\bf{n}}}}}$ is planar, the nanorods align as a function of the local shear, as illustrated. **b** The ${I}_{{zx}}$ spectrum measured at the center of the channel for the magnetic dipole (MD) (^5^D_0_-^7^F_1_) and electric dipole (ED) (^5^D_0_-^7^F_4_) transitions. The highlighted regions indicate the regions used for the area under the curve ratiometry. **c** Plots of the ratiometric ${k}_{{zx},{{\rm{MD}}}}$ and ${k}_{{zx},{{\rm{ED}}}}$ values. **d** Order parameter S (colormap and length of arrows) and the orientation of $\vec{{{{\bf{n}}}}}$ (arrows’ direction).

Let’s consider flowing a suspension of nanorods through a microfluidic channel with a square cross-section and a triangular obstacle as illustrated in Fig. 6a. At the middle height, $\vec{{{{\bf{n}}}}}$ is expected to be planar due to the symmetric shear conditions above and below the plane. Within this middle xy-plane, the nanorods align close to the stream lines and S depends on the local strength of the shear, such that the alignment is high at the walls but low near the center of the channel (Fig. 6a). To calculate the local alignment, a tomographic measurement of the three emission spectra ${I}_{{zx}},\,{I}_{z45^\circ }$ and ${I}_{{zy}}$ is performed with a pixel size of 1 μm^2^. Here, area-under-the-curve (AUC) ratiometry will be demonstrated, but spectral fitting can also be performed and yields similar results. In this approach, we consider the ratio between the AUC of two subpeaks for each type of dipole, referred to as ${k}_{{zx},{{\rm{MD}}}}$ and ${k}_{{zx},{{\rm{ED}}}}$ respectively, as indicated in Fig. 6b, c. Equation 11 can be written in terms of the intensity such that

12 $$\phi=\frac{1}{2}\left({\tan }^{-1}\varSigma+\frac{\pi }{2}\cdot \frac{\varSigma }{\left|\varSigma \right|}\cdot \left(\frac{\varGamma }{\left|\varGamma \right|}+1\right)\right)$$

where,

$$\Sigma=\frac{2\cdot {I}_{z45}-{I}_{{zx}}-{I}_{{zy}}}{{I}_{{zx}}-{I}_{{zy}}}\,,\,\Gamma={I}_{{zx}}-{I}_{{zy}}$$

As the three spectra are measured sequentially, normalization using the AUC of an unpolarized peak is applied to compensate for global intensity fluctuations. To optimize the sensitivity, we consider the AUC of only the first ED peak as this has the largest DOP (Fig. S7). Using the AUC of the ED1 peak for all three measured spectra thus allows to find the map of ϕ using Eq. 12. Equation 9 can be rewritten in terms of ${k}_{{zx},{{\rm{ED}}}}$ and the AUC of the subpeaks of the reference spectra such that:

13 $$S=\frac{1}{1-3\cdot {\sin }^{2}\theta \cdot{\cos }^{2}\phi }\cdot \frac{{I}_{\pi,{{\rm{ED}}}1}+2\cdot {I}_{\sigma,{{\rm{ED}}}1}-{k}_{{zx},{{\rm{ED}}}}\cdot ({I}_{\pi,{{\rm{ED}}}2}+2\cdot {I}_{\sigma,{{\rm{ED}}}2})}{{I}_{\pi,{{\rm{ED}}}1}-{I}_{\sigma,{{\rm{ED}}}1}-{k}_{{zx},{{\rm{ED}}}}\cdot ({I}_{\pi,{{\rm{ED}}}2}-{I}_{\sigma,{\rm{ED}}2})}$$

Using the assumption that θ = 90°, the map of ${k}_{{zx},{{\rm{ED}}}}$ (Fig. 6c) and the reference spectra (Fig. S14), S can thus be found through Eq. 13. To visualize the results, Fig. 6d shows a color map of S overlayed with arrows representing $\vec{{{{\bf{n}}}}}$. These results are consistent with our previous work and spectral fitting based on the three spectra gives comparable results (Fig. S15).

**Fig. 7:**
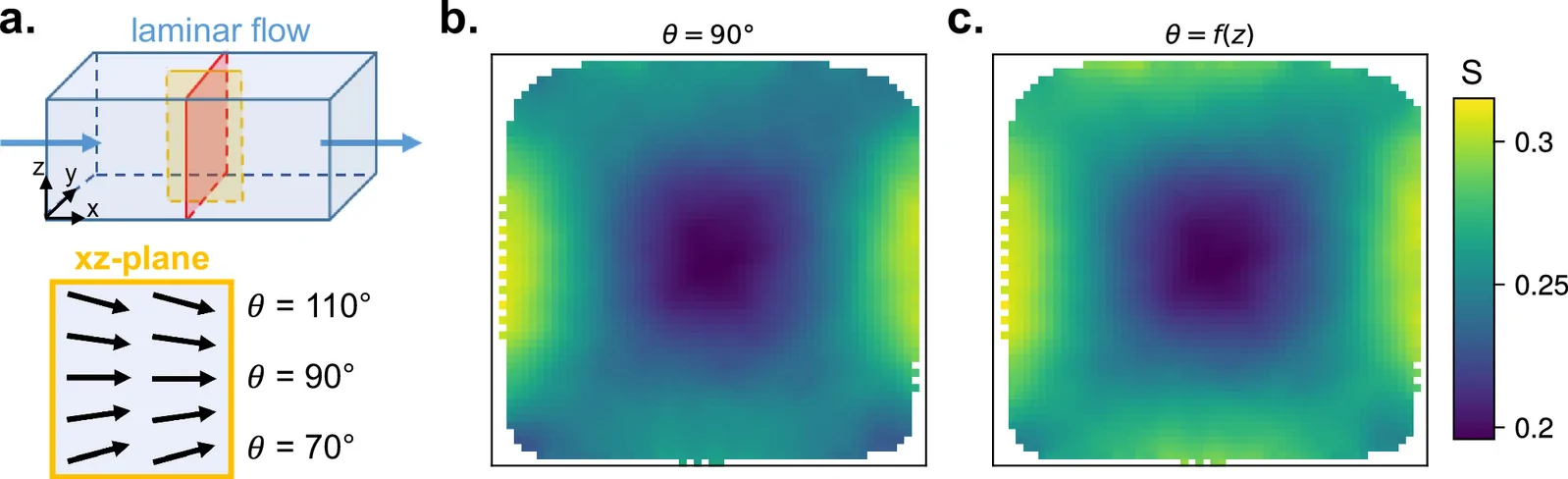
Correction of tilt angle in cross section of a microfluidic channel. **a** Schematic illustration of the microfluidic channel with a 50-by-50 μm^2^ cross section with a suspension of monazite LaPO_4_:Eu nanorods flowing at 10 nL/min. The director $\vec{{{{\bf{n}}}}}$ (indicated by the arrows) varies throughout the depth of the channel, and its polar angle θ is expected to change linearly^13^. **b, c** Calculated order parameter S at the cross section of the channel (indicated by red plane in a), based on the assumption that the $\theta \,$ is **b** 90° (planar) or **c** a linear function of the z-position such that $\theta \left(z\right)=\left(90^\circ -\Delta \theta \right)+z\cdot (2\cdot \Delta \theta /H)$ where $\Delta \theta$ is the change in θ between the center and the walls and H is the height of the channel.

In all our previous work regarding shearmetry, we simplified the system by assuming the director was fully planar throughout. However, even in the case of fully horizontal streamlines, such as a simple straight channel, the director will have a certain tilt depending on the local shear conditions, as illustrated in Fig. 7a. This difference between $\vec{{{{\bf{n}}}}}$ and the streamlines is well described in the fundamental theory of ellipsoidal particles under shear (flow)^34,35^. Based on the particle properties and flow conditions, we can estimate that this angle varies from +20° to -20° between the two walls (with 0° at the center) of the channel (described in the SI)^35^. When this effect is not considered, the resulting map of S is not symmetric, as S is much higher at the side walls than the top and bottom walls (Fig. 7b). With the method described here, it is easy to input any value for θ when calculating S in Eq. 13. Using a linear gradient of θ from 70° to 110° through the depth of the channel, we indeed retrieve a symmetrical S profile. For microfluidic shearmetry experiments, it is thus important to estimate the profile of θ(z) to guarantee the accuracy of the measurement.

## Discussion

This work builds on our previous demonstrations by developing a comprehensive framework for orientation tomography in both dilute and dense nanoparticle assemblies and by providing detailed experimental guidelines. Notably, evaluating the quality of the reference spectra by simulating an isotropic spectrum strongly improves the reproducibility and accuracy of the orientation analysis. We observed that both spectral fitting and AUC ratiometry give accurate and comparable orientation analysis results. The spectral curve fitting is generally more accurate in the case of low signal-to-noise ratios, but requires the use of a spectrometer, which is costly and results in large amounts of data. Instead, the AUC for each subpeak could be effectively calculated by placing appropriate filters in the detection path and capturing the intensities on photodetectors. Additionally, such filter-based measurements could also be implemented to widefield microscopy by splitting the detection path and capturing the AUC intensity images on different regions of a camera. Finally, the computation time of AUC ratiometry is significantly faster than the spectral fitting approach, which makes it a better candidate for live imaging.

Although the resolution (0.5 by 1 μm) is more than sufficient for tomographic imaging of the systems explored in this work, it is far from the theoretical resolution (0.14 μm by 0.3 μm). Aligning the excitation and emission planes remains experimentally challenging, and even small mismatches can significantly degrade resolution. This could be improved by using a motorized z-stage for the focusing lens and pinhole and implementing an iterative optimization algorithm. Additionally, we observe clear aberrations in the shape of the focused excitation beam. We experimentally observed that this is partly due to the flatness of the dichroic mirror and potentially due to under-filling the objective back aperture. Further improving the quality of the optical components and carefully controlling the alignment procedure should enhance the spatial resolution.

A main limitation which remains is the need for an assumption on either S or θ to solve the system. However, this is the case for all optical ensemble characterization techniques and to our knowledge there has not been a demonstration of simultaneous measurement of S and θ without any prior assumptions. This is due to the fact that for any dipole emission, a loss in order has a similar effect on the measured emission as an appearance of out-of-plane tilt. To resolve this, a second observation- or excitation angle could be introduced, but this is experimentally challenging. Nonetheless, our approach makes it possible to easily address systems with intermediate values of S and θ, which is not the case for other techniques whose models either assume perfect local order or a fixed planar or vertical alignment.

To conclude, this work provides a complete overview of both the experimental and theoretical foundations required to implement polarization-resolved spectroscopy of lanthanides for applications in orientation probing. We demonstrated its application for orientation tomography in LC systems and for order mapping in fluid flows (used for shearmetry), but the technique could easily be extended to other systems containing lanthanide-doped particles or molecules.

## Methods

### Preparation of Eu-doped nanoparticle samples

The NaYF_4_:Eu^3+^ nanorods used as single emitters for resolution measurements were synthesized according to the following protocol^1^. 45 mmol of NaOH dissolved in 6 mL of water was mixed with 15 mL of ethanol (EtOH) and 30 mL of oleic acid (OA) whilst stirring. A 4 mL aqueous solution of 0.95 mmol of Y(Cl)_3_·6H_2_O, 0.05 mmol of Eu(Cl)_3_·6H_2_O, and 10.2 mmol of NH_4_F was then added to the resulting mixture. The solution was then heated in an autoclave at 200 °C for 24 hours whilst stirring. Resulting nanorods were washed with water and EtOH, vacuum dried, and collected as a powder. To improve dispersion in polar solvents, a surface functionalization was performed by mixing 20 mg of NaYF_4_:Eu nanorods with native OA ligands were mixed with 2 mL of 0.2 M aqueous citrate solution, after which the solution was sonicated and centrifuged. This was repeated three times to exchange all OA with citrate and residual OA was removed by washing with EtOH and water. The resulting nanorods were dispersed in water and deposited on a microscope slide by spin coating.

Aqueous suspensions of LaPO_4_:Eu nanorods in both rhabdophane and monazite phase were obtained through two types of hydrothermal syntheses. Rhabdophane LaPO_4_:Eu nanorods were obtained according to the following protocol^19,20^. Aqueous solutions of (NH_4_)_2_HPO_4_ (100 mM), La(NO_3_)_3_·6H_2_0 (100 mM), Eu(NO_3_)_3_·H_2_0 (100 mM) were mixed at stoichiometric proportions corresponding to a 5% Eu-doping rate and heated at 160 °C for 3 hours in an autoclave. LaPO_4_:Eu nanorods in monazite phase were obtained through hydrothermal microwave synthesis^27^. Aqueous solutions of (NH_4_)_2_HPO_4_ (50 mM), La(NO_3_)_3_·6H_2_0 (50 mM), Eu(NO_3_)_3_·H_2_0 (50 mM) were mixed such that the final solution corresponded to a 20% excess of (NH_4_)_2_HPO_4_ and a 5% Eu-doping rate. The resulting suspension was heated in a microwave reactor under continuous stirring at maximum power at 160 °C for 2 hours. Both types of synthesized LaPO_4_:Eu nanoparticles were collected by centrifugation and re-dispersed in pH 2 water (HNO_3_ solution) through vortex mixing. The suspensions were then purified by dialysis for two days in aqueous HNO_3_ (pH = 2).

To obtain liquid crystal suspensions, synthesized nanoparticles are transferred to ethylene glycol (EG) using a rotary pump. The resulting solutions are left in glass vials until isotropic-nematic phase separation is completed. The nematic phase of these LC suspensions of LaPO_4_:Eu^3+^ in EG were used for obtaining the reference spectra and highly diluted suspensions ( < 0.1 vol%) of the isotropic phase for measuring the isotropic spectrum^19^. The assemblies of rhabdophane phase LaPO_4_:Eu^3+^ nanorods for demonstrating the 3D orientation tomography were generated by placing a drop of the biphasic (isotropic-nematic) LC between two coverslips separated by a 60 μm spacer. Microfluidic experiments were performed using a diluted isotropic suspension (0.9 vol%) of monazite phase LaPO_4_:Eu^3+^ in ethylene glycol.

### Optical measurements

Tomographic measurements of the polarized luminescence spectra were performed using the experimental setup shown in Fig. 2a. The optical setup includes an inverted confocal microscope (Nikon Eclipse Ti, Objective: magnification = 60x, NA = 1.49) equipped with a piezoelectric stage. A fiber-coupled monochromatic excitation laser (OXXIUS, *λ*_center_  =  394 nm) is used to induce Eu^3+^ emission. The dichroic mirror (Semrock FF470-Di01) reflects the excitation beam to the objective and transmits the collected emission to a prism mirror followed by the built-in tube lens (f = 200 mm). The 50 μm pinhole is mounted on a three axes translational stage to finely optimize the optical sectioning. A long pass filter (λ > 560 nm) eliminates the light originating from the excitation laser. The polarization state is selected using a λ/2 waveplate on a motorized rotating mount placed in front of a polarizing beam splitter with an additional linear polarizer placed in front of the spectrometer. The luminescence spectrum is recorded by a spectrometer (IsoPlane SCT-320, Teledyne Princeton Instruments) equipped with an electron multiplying CCD camera (ProEm + : 1600 EMCCD, Teledyne Princeton Instruments). An avalanche photodiode (APD - Thorlabs APD440A2) is used to perform luminescence intensity tomography. The scanning of the piezoelectric stage, rotation of the waveplate mount and acquisition of the spectra were synchronized by a central processor unit and software developed by Inscoper.

POM images of the LC assembly were taken on an Olympus BX51 microscope with crossed polarizers equipped with a CCD camera (Discovery plus DTA DX 1600E SN).

### Microfluidic experiments

Microfluidic channels were fabricated using a standard polydimethylsiloxane (PDMS) method. SYLGARD 184 silicone elastomer base and curing agent were thoroughly mixed in a weight ratio of 10:1, degassed and cast into the channel molds. After curing at 65 °C overnight, the PDMS and glass substrates were cleaned using a 60 s plasma treatment and stuck together to form the channels. They were then treated at 90 °C overnight to enhance the adhesion between the PDMS and the glass. Finally, a syringe pump (KD Scientific LEGATO 270) was used to perform the flow experiments at a constant flow rate.

### Reporting summary

Further information on research design is available in the Nature Portfolio Reporting Summary linked to this article.

## Supplementary information

Supplementary Information

Reporting Summary

Transparent Peer Review file

## Data Availability

The raw data generated in this study, including polarization-resolved spectral data, three-dimensional tomographic datasets, planar measurements, and reference spectra of rhabdophane and monazite LaPO₄:Eu^3+^, are openly available in the Zenodo repository under accession code https://doi.org/10.5281/zenodo.22638798^36^.

## Code availability

The data analysis was performed using Python scripts. Sample code and exemplary datasets can be accessed through the repository https://doi.org/10.5281/zenodo.17804430^37^.
