# Supplementary material for: Orientation tomography of lanthanide-doped nanoprobes by polarization-resolved micro-spectroscopy
Source: Nat Commun. 2026 Oct 14;17:11601. doi: 10.1038/s41467-026-78323-0 (PMC13751601; doi:10.1038/s41467-026-78323-0)
Supplement: Supplementary file 1 [file 41467_2026_78323_MOESM1_ESM.pdf]

Supporting Information

Orientation tomography of lanthanide-doped nanoprobes by polarization-resolved micro-spectroscopy

*Lilian Magermans, Marcello Bonetti, Thierry Gacoin and Jongwook Kim**

Contents

1. Derivation of the orientation analysis equations 2
2. Orientation equations for area under the curve (AUC) ratiometry 3
3. Calculation of homeotropic order parameter 5
4. Mirror angle discrimination for systems with known order parameter 6
5. Correcting birefringence distortions in thick samples 7
6. Simulation of POM images 9
7. Tilt angle approximation under flow 9
8. Supporting Figures 11
9. References 16

1. Derivation of the orientation analysis equations

For a single nanorod, the emission spectra measured at orthogonal polarizer axes can be expressed as a projection of the reference spectra onto the polarizer axis such that:

$$I_{zx}(\lambda) = I_\pi(\lambda) \cdot \sin^2\theta \cdot \cos^2\phi + I_\sigma(\lambda) \cdot \sin^2\theta \cdot \sin^2\phi + I_\alpha(\lambda) \cdot \cos^2\theta \qquad \text{(S1)}$$

$$I_{zy}(\lambda) = I_\pi(\lambda) \cdot \sin^2\theta \cdot \sin^2\phi + I_\sigma(\lambda) \cdot \sin^2\theta \cdot \cos^2\phi + I_\alpha(\lambda) \cdot \cos^2\theta \qquad \text{(S2)}$$

where I_{zx} and I_{zy} are the emission spectra measured with the analyzer parallel to the x and y-axis respectively and θ, ϕ are the polar and azimuthal angles with respect to the z-axis, as illustrated in Figure S1.

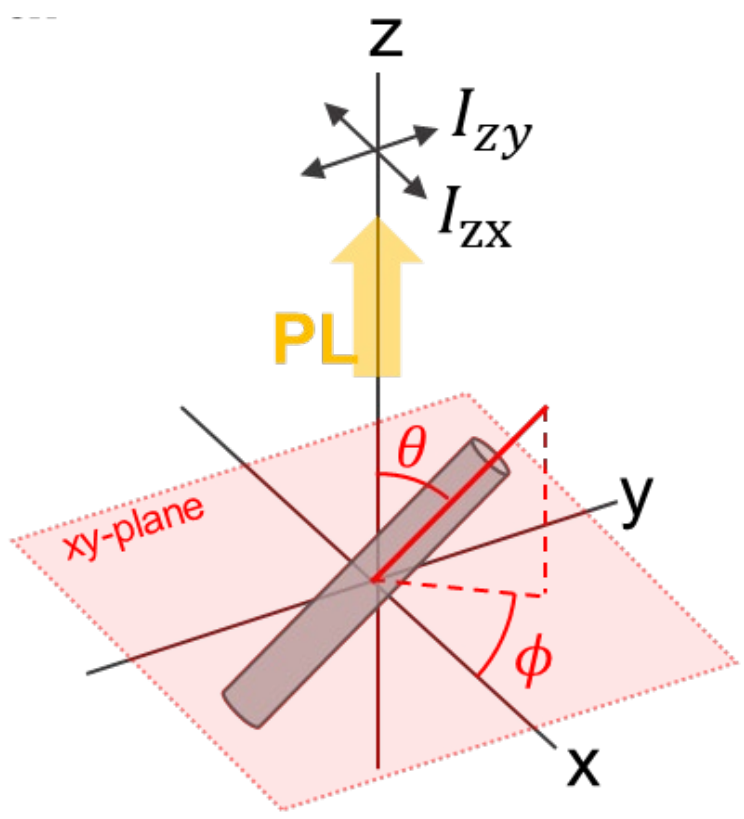

Figure S1: Schematic illustration of the orientation of a single nanorod. The orientation of a single nanorod with respect to the laboratory frame where the xy-plane is the observation plane of the microscope and the z-axis is the direction of the photoluminescence collection.

For an ensemble of many nanorods, its orientation is quantified by the average orientation, the director $\vec{\mathbf{n}}(\theta, \phi)$ and its order parameter S (see equation 6). Each individual nanorod within the ensemble has a certain orientation with respect to $\vec{\mathbf{n}}$, indicated by (θ_S, ϕ_S) as illustrated in Figure 3b. The polarized emission of a single particle within the ensemble can be expressed as the projection of the three reference spectra onto the axis parallel and perpendicular to $\vec{\mathbf{n}}$, such that

$$I_\parallel(\lambda) = \langle I_\pi(\lambda) \cdot \cos^2\theta_s + I_\sigma(\lambda) \cdot \sin^2\theta_s \cdot \sin^2\phi_s + I_\alpha(\lambda) \cdot \sin^2\theta_s \cdot \cos^2\phi_s \rangle \qquad \text{(S3)}$$

$$I_\perp(\lambda) = \langle I_\pi(\lambda) \cdot \sin^2\theta_s \cdot \sin^2\phi_s + I_\sigma(\lambda) \cdot \cos^2\theta_s + I_\alpha(\lambda) \cdot \sin^2\theta_s \cdot \cos^2\phi_s \rangle \qquad \text{(S4)}$$

where $\langle \ldots \rangle$ represent an average over all nanorods within the ensemble. Note that $I_\alpha(\lambda)$ is identical to either $I_\pi(\lambda)$ or $I_\sigma(\lambda)$ for magnetic dipole (MD) or electric dipole (ED) transitions respectively (equations 1-2). Additionally, the cylindrical symmetry around $\vec{\mathbf{n}}$ dictates that $\langle \cos^2\phi_s \rangle = \langle \sin^2\phi_s \rangle = \frac{1}{2}$ which allows to simplify equations S3-4 for both MD and ED such that

$$I_{\parallel,\mathrm{MD}}(\lambda) = \frac{I_{\pi,\mathrm{MD}}(\lambda)}{2} \cdot (1 + \langle \cos^2\theta_s \rangle) + \frac{I_{\sigma.\mathrm{MD}}(\lambda)}{2} \cdot (1 - \langle \cos^2\theta_s \rangle) \qquad \text{(S5)}$$

$$I_{\perp,\mathrm{MD}}(\lambda) = I_{\pi,\mathrm{MD}}(\lambda) \cdot (1 - \langle \cos^2\theta_s \rangle) + I_{\sigma,\mathrm{MD}}(\lambda) \cdot \langle \cos^2\theta_s \rangle \qquad \text{(S6)}$$

$$I_{\parallel,\mathrm{ED}}(\lambda) = I_{\pi,\mathrm{ED}}(\lambda) \cdot \langle \cos^2\theta_s \rangle + I_{\sigma,\mathrm{ED}}(\lambda) \cdot (1 - \langle \cos^2\theta_s \rangle) \qquad \text{(S7)}$$

$$I_{\perp,\mathrm{ED}}(\lambda) = \frac{I_{\pi,\mathrm{ED}}(\lambda)}{2} \cdot (1 - \langle\cos^2\theta_s\rangle) + \frac{I_{\sigma,\mathrm{ED}}(\lambda)}{2} \cdot (1 + \langle\cos^2\theta_s\rangle) \tag{S8}$$

The emission measured with respect to the laboratory frame, is then a projection of $I_{\parallel}(\lambda)$ and $I_{\perp}(\lambda)$ onto the polarizer axis such that

$$I_{zx,\mathrm{MD}}(\lambda) = I_{\parallel,\mathrm{MD}}(\lambda) \cdot (\sin^2\theta \cdot \cos^2\phi + \cos^2\theta) + I_{\perp,\mathrm{MD}}(\lambda) \cdot \sin^2\theta \cdot \sin^2\phi \tag{S9}$$

$$I_{zy,\mathrm{MD}}(\lambda) = I_{\parallel,\mathrm{MD}}(\lambda) \cdot (\sin^2\theta \cdot \sin^2\phi + \cos^2\theta) + I_{\perp,\mathrm{MD}}(\lambda) \cdot \sin^2\theta \cdot \cos^2\phi \tag{S10}$$

$$I_{zx,\mathrm{ED}}(\lambda) = I_{\parallel,\mathrm{ED}}(\lambda) \cdot \sin^2\theta \cdot \cos^2\phi + I_{\perp,\mathrm{ED}}(\lambda) \cdot (\sin^2\theta \cdot \sin^2\phi + \cos^2\theta) \tag{S11}$$

$$I_{zy,\mathrm{ED}}(\lambda) = I_{\parallel,\mathrm{ED}}(\lambda) \cdot \sin^2\theta \cdot \sin^2\phi + I_{\perp,\mathrm{ED}}(\lambda) \cdot (\sin^2\theta \cdot \cos^2\phi + \cos^2\theta) \tag{S12}$$

where $I_{zx}(\lambda)$ and $I_{zy}(\lambda)$ are the emission spectra measured with the analyzer parallel to the x and y-axis respectively. These equations are analogous to those for single particles (equations S1-S2), with $I_{\parallel}(\lambda)$ and $I_{\perp}(\lambda)$ replacing $I_{\pi}(\lambda)$ and $I_{\sigma}(\lambda)$ respectively. Substituting equations S5-S8 into equations S9-12 and considering the definition of S (equation 6) yields the complete expressions for $I_{zx}(\lambda)$ and $I_{zy}(\lambda)$ described by equations 7-10 in the main text.

Additionally, we can define the expressions of the emission measured with the analyzer at 45° with respect to the x-axis as

$$I_{z45,\mathrm{MD}}(\lambda) = I_{\parallel,\mathrm{MD}}(\lambda) \cdot \left(\sin^2\theta \cdot \cos^2\left(\phi - \frac{\pi}{4}\right) + \cos^2\theta\right) + I_{\perp,\mathrm{MD}}(\lambda) \cdot \sin^2\theta \cdot \sin^2\left(\phi - \frac{\pi}{4}\right) \tag{S13}$$

$$I_{z45,\mathrm{ED}}(\lambda) = I_{\parallel,\mathrm{ED}}(\lambda) \cdot \sin^2\theta \cdot \cos^2\left(\phi - \frac{\pi}{4}\right) + I_{\sigma,\mathrm{ED}}(\lambda) \cdot \left(\sin^2\theta \cdot \sin^2\left(\phi - \frac{\pi}{4}\right) + \cos^2\theta\right) \tag{S14}$$

Using the trigonometric relationships $\cos^2\left(x - \frac{\pi}{4}\right) = \frac{1}{2} + \frac{1}{2} \cdot \sin 2x$ and $\sin^2\left(x - \frac{\pi}{4}\right) = \frac{1}{2} - \frac{1}{2} \cdot \sin 2x$ these equations can be simplified to

$$I_{z45,\mathrm{MD}}(\lambda) = \frac{I_{\parallel,\mathrm{MD}}(\lambda)}{2} \cdot (\sin^2\theta \cdot (1 + \sin 2\phi) + 2\cos^2\theta) + \frac{I_{\perp,\mathrm{MD}}(\lambda)}{2} \cdot \sin^2\theta \cdot (1 - \sin 2\phi) \tag{S15}$$

$$I_{z45,\mathrm{ED}}(\lambda) = \frac{I_{\parallel,\mathrm{ED}}(\lambda)}{2} \cdot \sin^2\theta \cdot (1 + \sin 2\phi) + \frac{I_{\perp,\mathrm{ED}}(\lambda)}{2} \cdot (\sin^2\theta \cdot (1 - \sin 2\phi) + 2\cos^2\theta) \tag{S16}$$

Considering equations S9-12, it can be deduced that I_{z45} can be expressed in terms of I_{zx} and I_{zy}, for both MD and ED, such that

$$2 \cdot I_{z45}(\lambda) = I_{zx}(\lambda) + I_{zy}(\lambda) + \left(I_{zx}(\lambda) - I_{zy}(\lambda)\right) \cdot \tan(2\phi) \tag{S17}$$

2. Orientation equations for area under the curve (AUC) ratiometry

Instead of using the full measured spectra to solve the orientation equations, it can be advantages to use singular intensity values. To eliminate the impact global intensity fluctuations, this can be done by taking the ratio of the area under the curve (AUC) of two subpeaks within the transition of interest. For a given spectrum $I(\lambda)$ and a dipole D (either MD or ED) we define the ratiometric value $k_D = I_{D1}/I_{D2}$, where I_{D1} and I_{D2} are the AUC of two oppositely polarized subpeaks within the same kind of dipole transition. Similarly, we can replace the reference spectra $I_{\pi}(\lambda)$ and $I_{\sigma}(\lambda)$ by the AUC values of the same subpeaks denoted as $I_{\pi,D1}, I_{\pi,D2}, I_{\sigma,D1}$ and $I_{\sigma,D2}$, as illustrated in Figure S2.

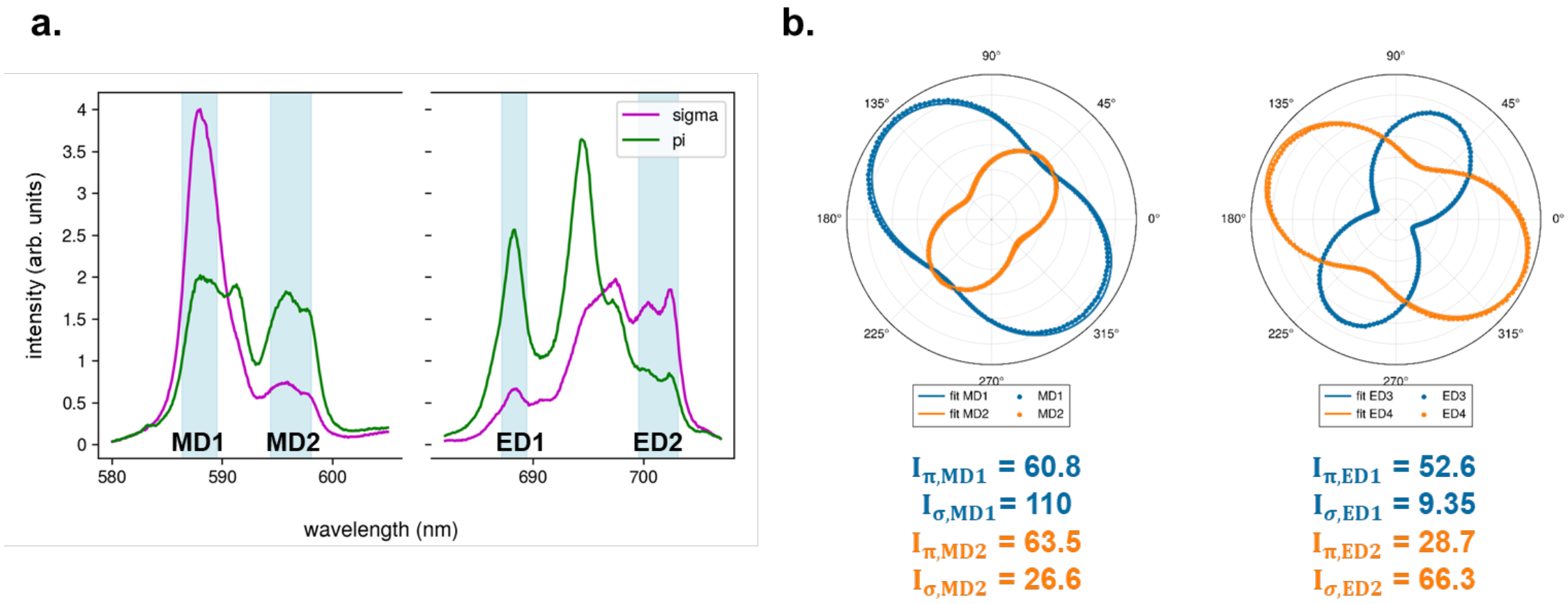

Figure S2: Area under the curve (AUC) for the reference spectra. a) Reference spectra of rhabdophane phase $LaPO_4$:Eu (see Figure 1d). The blue regions signify the wavelength ranges used to calculate the AUC of the different subpeaks indicated as MD1, MD2, ED1 and ED2. **b)** Calculated AUC values as a function of the orientation of the analyzer based on the measurement in Figure 1b. The director orientation of the reference sample does not need to be known, the reference $I_{\pi,D1}, I_{\pi,D2}, I_{\sigma,D1}$ and $I_{\sigma,D2}$ (where D is MD or ED) values are found by fitting the points to the dipolar model in equation 3.

As an example, we take the case of a known order parameter S and rewrite the expressions of for the I_{zx} spectrum (equations 7 and 9) to solve for the unknown values $\sin^2\theta$ and $\cos^2\phi$ in terms of the ratiometric values $k_{zx,\mathrm{MD}}$ and $k_{zx,\mathrm{ED}}$.

$$\sin^2\theta\cdot(1-\cos^2\phi)=\frac{(S+2)\cdot\left(I_{\pi,\mathrm{MD1}}-k_{zx,\mathrm{MD}}\cdot I_{\pi,\mathrm{MD2}}\right)+(1-S)\cdot\left(I_{\sigma,\mathrm{MD1}}-k_{zx,\mathrm{MD}}\cdot I_{\sigma,\mathrm{MD2}}\right)}{3S\cdot\left(I_{\pi,\mathrm{MD1}}-I_{\sigma,\mathrm{MD1}}-k_{zx,\mathrm{MD}}\cdot\left(I_{\pi,\mathrm{MD2}}-I_{\sigma,\mathrm{MD2}}\right)\right)}\tag{S18}$$

$$\sin^2\theta\cdot\cos^2\phi=\frac{(1-S)\cdot\left(I_{\pi,\mathrm{ED1}}-k_{zx,\mathrm{ED}}\cdot I_{\pi,\mathrm{ED2}}\right)+(S+2)\cdot\left(I_{\sigma,\mathrm{ED1}}-k_{zx,\mathrm{ED}}\cdot I_{\sigma,\mathrm{ED2}}\right)}{3S\cdot\left(-I_{\pi,\mathrm{ED1}}+I_{\sigma,\mathrm{ED1}}+k_{zx,\mathrm{ED}}\cdot\left(I_{\pi,\mathrm{ED2}}-I_{\sigma,\mathrm{ED2}}\right)\right)}\tag{S19}$$

This gives two equations with two unknowns, which allows to find a unique solution. We can easily sum equation S18 and S19 to get an expression for $\sin^2\theta$ and then use this value in either of the equations to solve for $\cos^2\phi$. Figure S3 shows a demonstration using the same data as Figure 4.

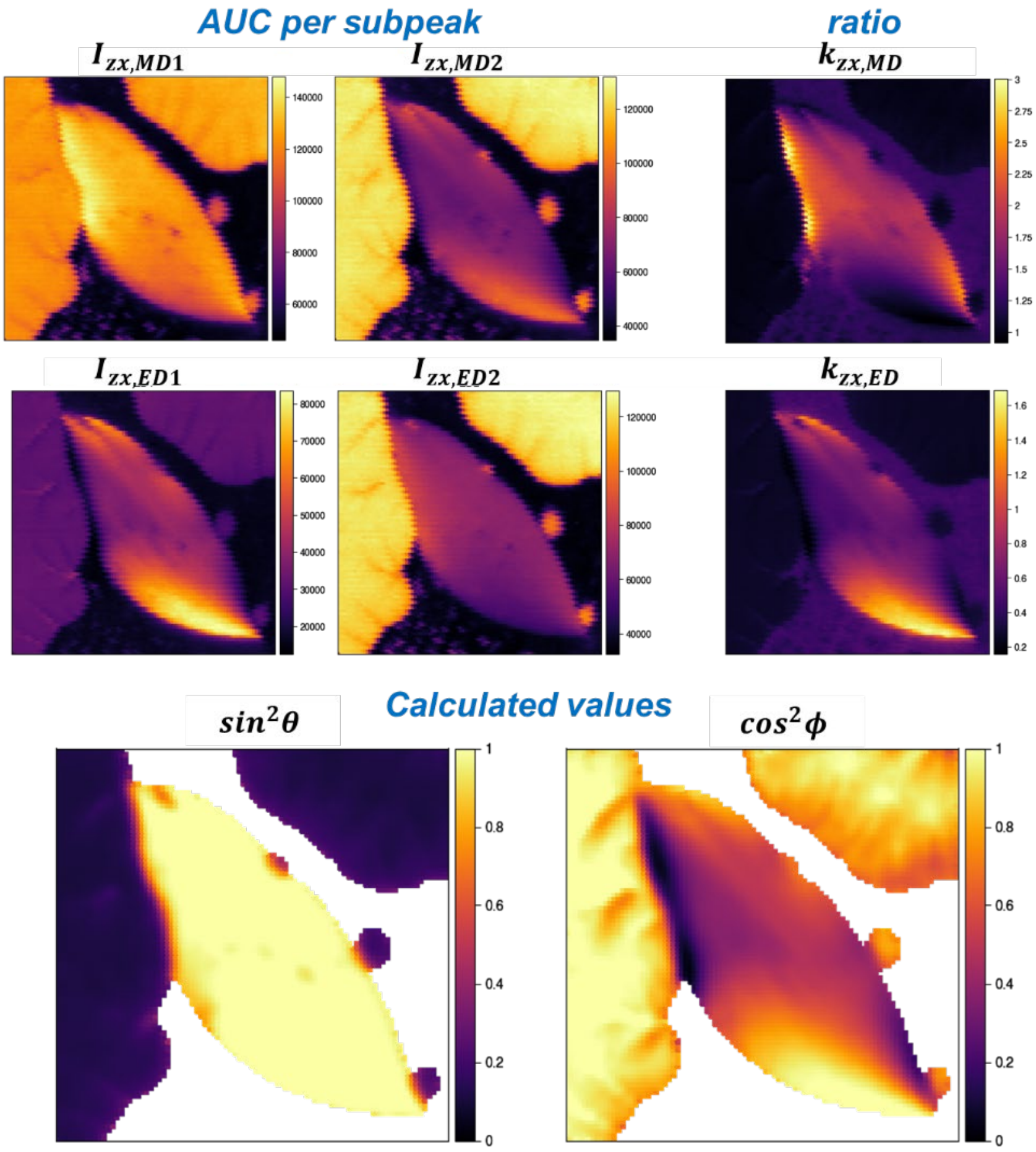

Figure S3: Orientation calculated based on AUC analysis using the I_{zx} spectrum. The AUC is calculated by integrating the subpeaks indicated in Figure S2a followed by the corresponding ratiometric k values. Then $\sin^2\theta$ and $\cos^2\phi$ are calculated based using equations S18-S19 and the reference.

3. Calculation of homeotropic order parameter

We previously demonstrated that the homeotropic order parameter S_{homeo}, for systems with a vertical director, can be spectroscopically measured without the need of a polarizer.[1] The spectrum collected without an analyzer is the sum of two orthogonally polarized spectra such that $I_z(\lambda) = I_{zx}(\lambda) + I_{zy}(\lambda)$. Assuming a vertical director (so θ = 0°) and considering expressions 7-10 we obtain

$$\frac{2}{3} \cdot I_{z,\text{MD}}(\lambda) = I_\pi(\lambda) \cdot (2 + S_{\text{homeo}}) + I_\sigma(\lambda) \cdot (1 - S_{\text{homeo}}) \tag{S20}$$

$$\frac{2}{3} \cdot I_{z,\text{ED}}(\lambda) = I_\pi(\lambda) \cdot (1 - S_{\text{homeo}}) + I_\sigma(\lambda) \cdot (2 + S_{\text{homeo}}) \tag{S21}$$

A spectral fitting (or AUC ratiometry) can then be used to find S_{homeo}, as shown in Figure S4.

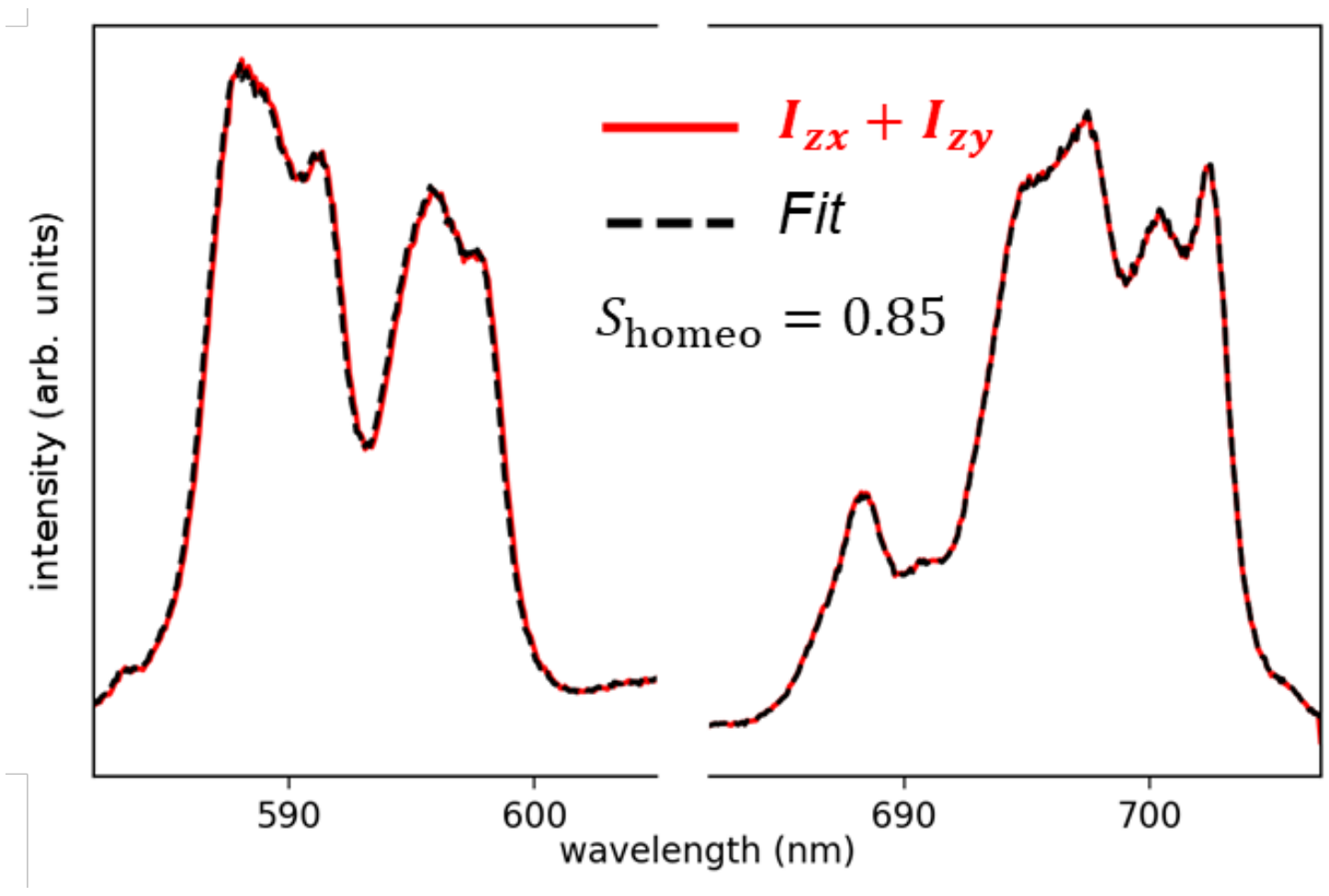

Figure S4: Spectral fit to determine the homeotropic (vertical director) order parameter S_{homeo}. The sum of the $I_{zx}(\lambda) + I_{zy}(\lambda)$ spectra recorded close to the substrate in the flower domain (Figure 4) is fit using expressions S20 and S21.

4. Mirror angle discrimination for systems with known order parameter

Although equation 12 directly provides ϕ, it requires a normalization which may be difficult to achieve. Where possible, it is better to first determine the values of $\sin^2\theta$ and $\cos^2\phi$. To eliminate the mirror angle of $|\phi|$, the $I_{z45°}(\lambda)$ is then used either by spectral fitting or AUC ratiometry. In the spectral fitting approach, equations S15 and/or S16 are fit to the measured $I_{z45°}(\lambda)$ spectra with $\sin^2\theta$ and $\cos^2\phi$ as fixed input values, such that ϕ can be either $\pm\cos^{-1}(\sqrt{\cos^2\phi})$. For the AUC approach, we combine the expressions for $I_{zx,\text{MD}}(\lambda)$ and $I_{z45°,\text{MD}}(\lambda)$, equations 7 and S15 respectively, in terms of the ratiometric k-values to find

$$\tan(2\phi) = \frac{(\mathrm{A} - k_{z45°,\text{MD}} \cdot \Gamma) \cdot (k_{zx,\text{MD}} \cdot \mathrm{E} - \mathrm{B})}{(k_{z45°,\text{MD}} \cdot \mathrm{E} - \mathrm{B}) \cdot (\mathrm{A} - k_{zx,\text{MD}} \cdot \Gamma)} \qquad \text{(S22)}$$

where

$$\mathrm{A} = \frac{S}{2} \cdot (I_{\pi,\text{MD1}} + I_{\sigma,\text{MD1}}) + (1 - \sin^2\theta) \cdot I_{\pi,\text{MD1}}$$

$$\mathrm{B} = \frac{\mathrm{S}}{2} \cdot (I_{\pi,\text{MD1}} - I_{\sigma,\text{MD1}})$$

$$\Gamma = \frac{S}{2} \cdot (I_{\pi,\text{MD2}} + I_{\sigma,\text{MD2}}) + (1 - \sin^2\theta) \cdot I_{\pi,\text{MD1}}$$

$$\mathrm{E} = \frac{S}{2} \cdot (I_{\pi,\text{MD2}} - I_{\sigma,\text{MD2}})$$

The value of ϕ can thus be directly determined from expression S22 if the values of $\sin^2\theta$ and S are known.

For now, there is no spectroscopic method for discriminating between $\pm\,\theta$, but knowledge of the boundary conditions can help solve this issue. For example, the LC system discussed in this work has planar anchoring at the nematic-isotropic interface, meaning the director should be tangential to the interface.

5. Correcting birefringence distortions in thick samples

We can model the nematic LC as an ideal linear retarder whose fast axis (parallel to the director) is defined as the x-axis such that its Jones matrix:

$$\mathrm{J} = \begin{bmatrix} 1 & 0 \\ 0 & e^{i\delta} \end{bmatrix} \tag{S23}$$

where δ is the phase retardation as defined by:

$$\delta \approx \frac{2\pi}{\lambda} \cdot t \cdot \Delta n_{\mathrm{app}} \tag{S24}$$

where λ is the wavelength of the illumination light, t is the sample thickness, Δn_{app} is the apparent birefringence and θ the obtained polar angle of the director.[2]

We then describe a linearly polarized PL emission component in terms of the angle between its polarization axis and the fast axis of the LC ψ such that:

$$\mathrm{E_{in}} = \begin{bmatrix} \cos\psi_{\mathrm{in}} \\ \sin\psi_{\mathrm{in}} \end{bmatrix} \tag{S25}$$

The output then becomes

$$\mathrm{E_{out}} = \mathrm{JE_{in}} = \begin{bmatrix} \cos\psi_{\mathrm{in}} \\ e^{i\delta}\sin\psi_{\mathrm{in}} \end{bmatrix} \tag{S26}$$

For arbitrary retardance, the output is generally elliptically polarized. The orientation of the polarization ellipse is given by

$$\tan 2\psi_{\mathrm{out}} = \frac{\sin 2\psi_{\mathrm{in}} \cos\delta}{\cos 2\psi_{\mathrm{in}}} \tag{S27}$$

Such that

$$\psi_{\mathrm{out}} = \frac{1}{2}\mathrm{atan2}(\sin 2\psi_{\mathrm{in}} \cos\delta, \cos 2\psi_{\mathrm{in}}) \tag{S28}$$

where $\mathrm{atan2}(y, x)$ is the two-argument inverse tangent which returns the angle in the correct quadrant. From equation S28 we see that if ψ_{in} is 0 or a multiple of π/2, then $\Psi_{\mathrm{out}} = \psi_{\mathrm{in}}$. Therefore, if the polarization axis (equivalently the long axis of the polarization ellipse) of the propagating light is parallel or perpendicular to the fast axis of the LC, it’s orientation will not be affected whilst propagating through the LC.

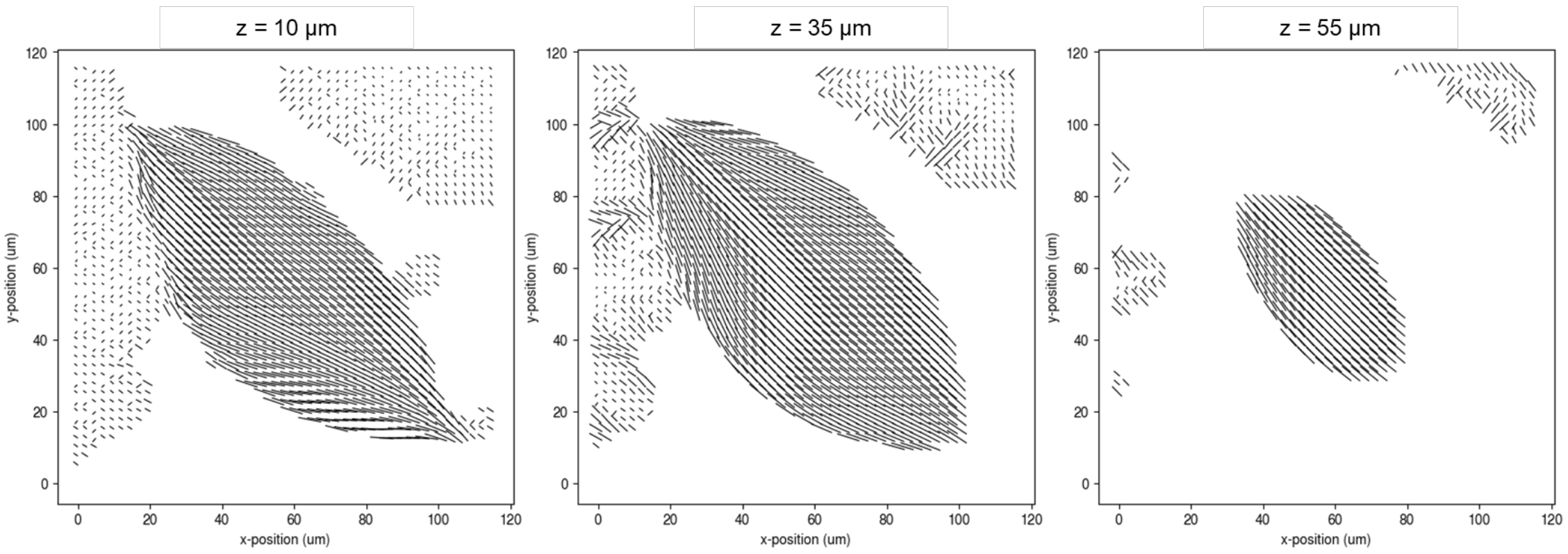

Figure S5: **Director profile at 3 different xy-planes corresponding to Figure 5e**.

In the case of the lanthanide emission discussed here, the emission dipoles produce linearly polarized emission whose axes are parallel and perpendicular to the nanoparticles. Therefore, if the orientation of the nanorods is (close to) uniform throughout the depth of the sample, no rotation of the PL polarization will be observed. This is indeed what we observed in the nematic LC samples. As shown in Figure S5, the measured director in the tactoid is largely uniform throughout the depth of the sample, and we thus do not observe any noticeable rotation in the polarization axis. In most colloidal LCs the twisting of the director (rotation across planes) is energetically unfavorable and we therefor do not expect significant apparent rotation of the polarization orientation measured with a linear analyzer. However, when working with chiral (nematic) liquid crystals or other dense assemblies whose director field rotates within the observation plane through the depth of the sample, the effect of the birefringence can be substantial and should be taken into consideration.

Let us consider a measured director at a fixed x,y position throughout the depth of the sample described by the orientation $(\theta(z), \phi(z))$ as calculated by the spectroscopic approach presented in this work. We consider that z_0 is the bottom plane, such that no distortions due to birefringence affect its measurement and thus the correct azimuthal orientation would simply be $\phi_{\text{corr}}(z_0) = \phi(z_0)$. However, the measured orientation at the subsequent plane z_1 is affected by the PL emission propagating through plane z_0. The apparent birefringence of plane z_0 can be approximated as a function of the director orientation and order parameter S such that

$$\Delta n_{\text{app},z_0} = \Delta n \cdot S \cdot \sin^2 \theta(z_0) \tag{S29}$$

where Δn is the intrinsic birefringence for perfectly aligned particles. The retardance of plane z_0 can then be expressed using equation S24 such that

$$\delta_{z0} \approx \frac{2\pi}{\lambda_{\text{PL}}} \cdot (z_1 - z_0) \cdot \Delta n \cdot S \cdot \sin^2 \theta(z_0) \tag{S30}$$

The measured orientation $\phi(z_1)$ is affected by the birefringence of the underlying layer z_0. The polarization ellipse exits plane z_0 at a relative angle $\Psi_{\text{out}} = \phi(z_1) - \phi_{\text{corr}}(z_0)$. We want to find its original relative orientation Ψ_{in}, which can be done by rearranging equation S28 such that

$$\psi_{\text{in}} = \frac{1}{2}\text{atan2}\left(\frac{\sin 2\psi_{\text{out}}}{\cos\delta}, \cos 2\psi_{\text{out}}\right) \quad \text{(S31)}$$

Finally, we can convert the relative angle back to the laboratory frame by

$$\phi_{\text{corr}}(z_1) = \phi_{\text{corr}}(z_0) + \Psi_{\text{in}} \quad \text{(S32)}$$

This correction can then be applied throughout the whole volume by evaluating the expected distortion plane by plane from the bottom up (for an inverted microscope).

6. Simulation of POM images

Based on the tomographic director maps obtained with the spectroscopic analysis, POM images can be reconstructed. The relative intensity transmitted between crossed polarizers for an aligned ensemble is

$$\frac{I(\psi)}{I_0} = \sin^2\big(2 \cdot (\phi - \psi)\big) \cdot \sin^2\frac{\delta}{2} \quad \text{(S33)}$$

where ψ is the orientation of the crossed polarizers with respect to the x-axis, ϕ the obtained azimuthal angle of the director and δ the phase retardation. Taking into account the impact of the tilt of the director, δ can be approximated as

$$\delta \approx \frac{2\pi}{\lambda} \cdot t \cdot \Delta n \cdot \sin^2\theta \quad \text{(S34)}$$

where λ is the wavelength of the illumination light, t is the sample thickness (or height of a voxel), Δn is the birefringence and θ the obtained polar angle of the director.[3] Using these expressions, the relative intensity can be calculated for each voxel in the measurement and an image is generated by adding the contributions of all z-planes. The images shown in Figure 5f-g are thus generated by calculating $I(\psi)$ for $\psi = 0°$ and $\psi = 45°$ respectively.

7. Tilt angle approximation under flow

To estimate the out-of-plane tilt of the director in a microfluidic channel, we combine the theory describing anisotropic particles under laminar flow with corresponding numerical simulations.

The collective orientation of the nanorods within the flowing suspension is determined by the competition of Brownian motion (random orientation) and the hydrodynamic forces (alignment with the streamlines) imposed by the flow conditions.[4] The fundamental Peterlin and Stuart theory, was initially developed to describe the stress-optical behavior of anisotropic suspensions, namely the birefringence observed between crossed polarizers.[5] In the classical experiment of a suspension confined between two counter rotating cylinders, the extinction angle χ is the angle between the polarizer and the dark axis observed between crossed polarizers. More generally, χ is related to the angle between the (tangent of the) streamline and the local director $\vec{n}$, such that $\chi = 45°$ when $\vec{n}$ is parallel to the streamline. Numerical evaluations done by Scheraga *et al.* show χ as a function of the parameter α, describing the ratio of the local velocity gradient G and the rotary diffusion coefficient Ω.[4] The orientation of the director with respect to the streamline, as well as the order parameter, can thus be

determined numerically if G and Ω are known. We will focus here on the director orientation, but have previously shown that experimental measurements of S as a function of γ also match the theoretical curves.

For the numerical simulations, the three-dimensional laminar flow (stationary) module of COMSOL was used with the following parameters: channel cross-section 50-by-50 μm, fully developed flow with flow rate 1.67e-13 m^3/s (10 nl/min) at the inlet, atmospheric pressure at the outlet. Although we have previously shown that the nanorods suspensions are shear thinning, within the flow regime shown here, the material can be approximated as Newtonian ethylene glycol. The COMSOL model can be easily adapted to consider the shear thinning behavior by creating a function for the viscosity following our reported measured values.[6] The resulting simulation (Figure S6), shows a linear dependency of the shear rate γ, varying from roughly 12 s^{-1} at the walls to 0 at the center of the channel.

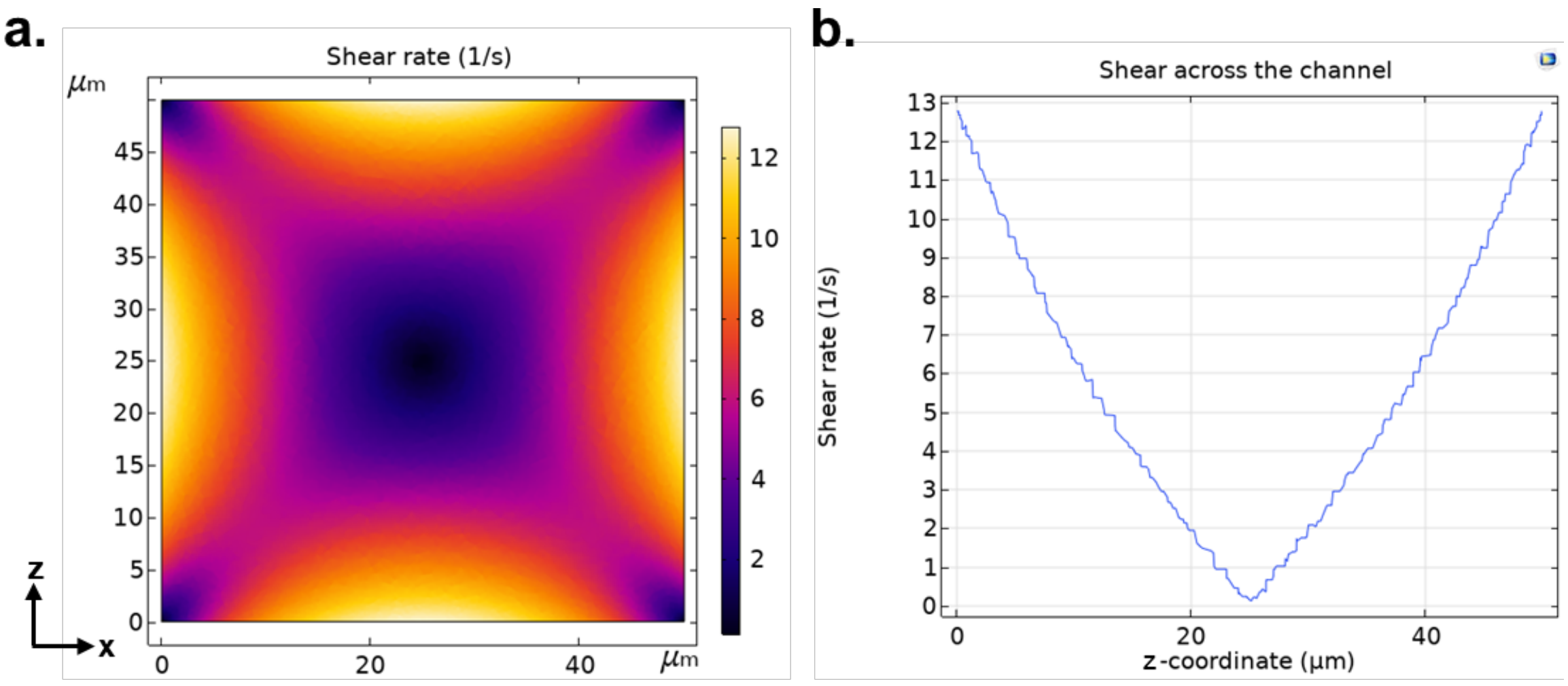

Figure S6 Numerical simulation of laminar flow through a square channel. Simulations are performed in COMSOL on a 3D channel with 50 μm by 50 μm square cross section. **a)** Plot of the shear rate (flow perpendicular to the plane shown). **b)** Plot of the shear rate across the depth of the channel at the center x-coordinate.

The diffusion coefficient of the monazite phase LaPO4:Eu suspension is approximated as $\Omega \approx$ 2 s^{-1} based on previous measurements. Combining this with the numerical simulations of the flow conditions, $\alpha \approx 6$ at the walls and 0 at the center. Based on the theoretical $\chi(\alpha)$ curve, this means χ varies from roughly 25° to 45°, corresponding to an angle of 0 to 20° between $\vec{n}$ and the streamline.[4] Considering that the streamlines are horizontal for a straight channel and taking into account the symmetry with respect to the center xy-plane, this means θ varies linearly from 70° to 110° from the bottom to the top of the channel.

8. Supporting Figures

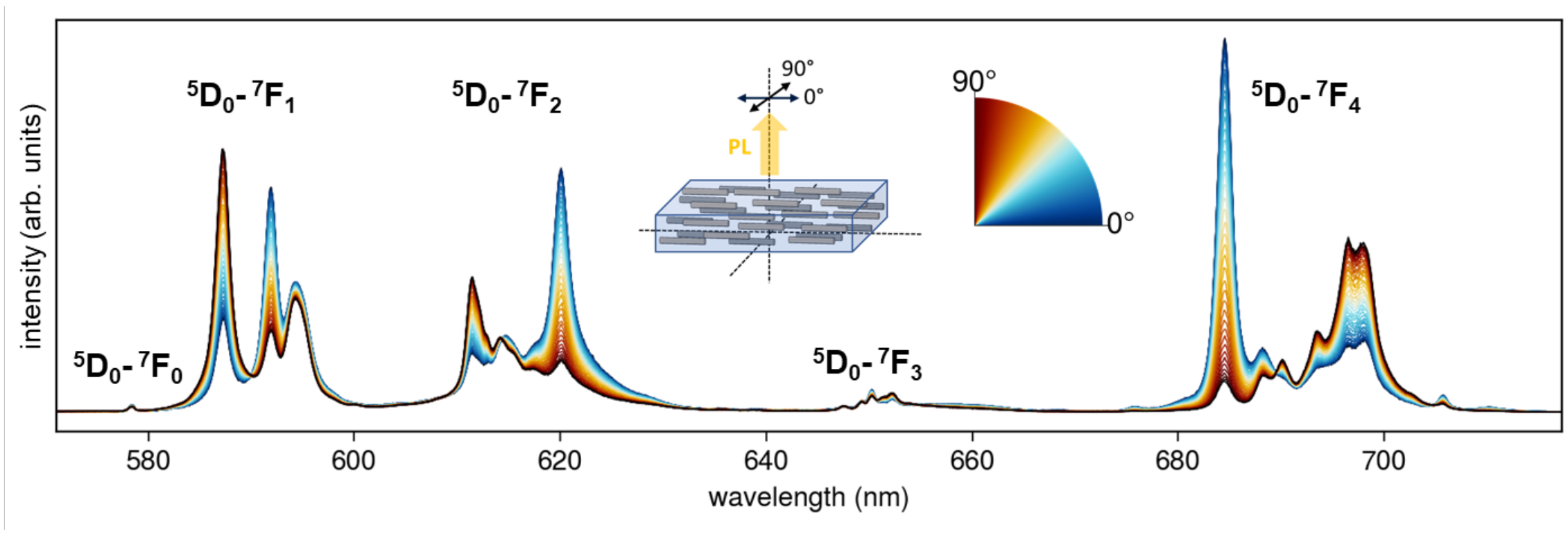

Figure S7: Polarized emission of monoclinic (monazite) $LaPO_4$:Eu nanorods. Polarized emission spectra measured in a dense, planar liquid crystal (LC) assembly when rotating the analyzer from 0° to 90° with respect to the horizontal alignment axis.

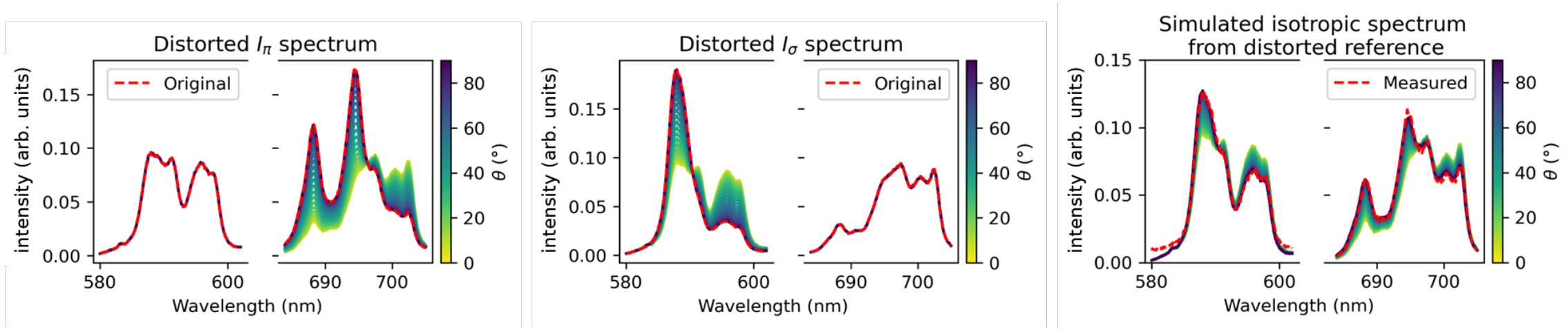

Figure S8: **Simulated impact of the director tilt θ of the reference sample on the obtained reference spectra** I_σ, I_π and simulated isotropic spectrum I_{iso} assuming $S = 1$. The red dashed line represents the original experimentally measured I_π , I_σ and I_{iso} respectively.

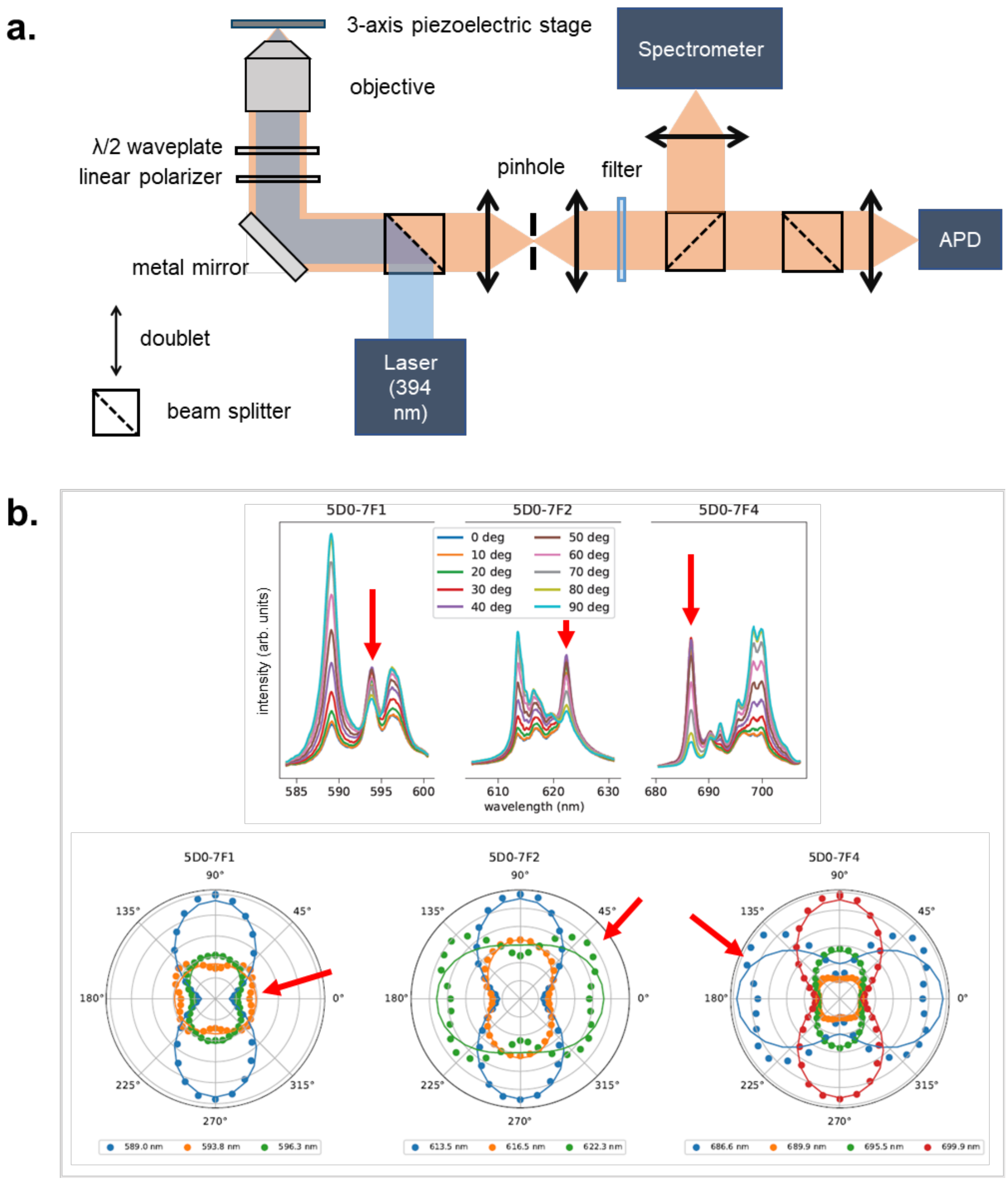

Figure S9: Effect of metallic mirror on the measured polarized emission spectra. a) Schematic illustration of the optical setup causing polarization distortions. The setup is similar to the one in Figure 2a, but here the polarization state is selected with a λ/2 waveplate followed by a linear polarizer placed right behind the objective and a metal mirror is used instead of a prism mirror. **b)** Polarized emission spectra collected in a dense, planar LC of monazite $LaPO_4$:Eu. For certain subpeaks (indicated by red arrows), the angular dependency of the intensity does not follow the expected dipolar model, but rather appears to have a butterfly shape. We attribute this to the metallic mirror, which effects the polarization state, similarly to a linear polarizer.

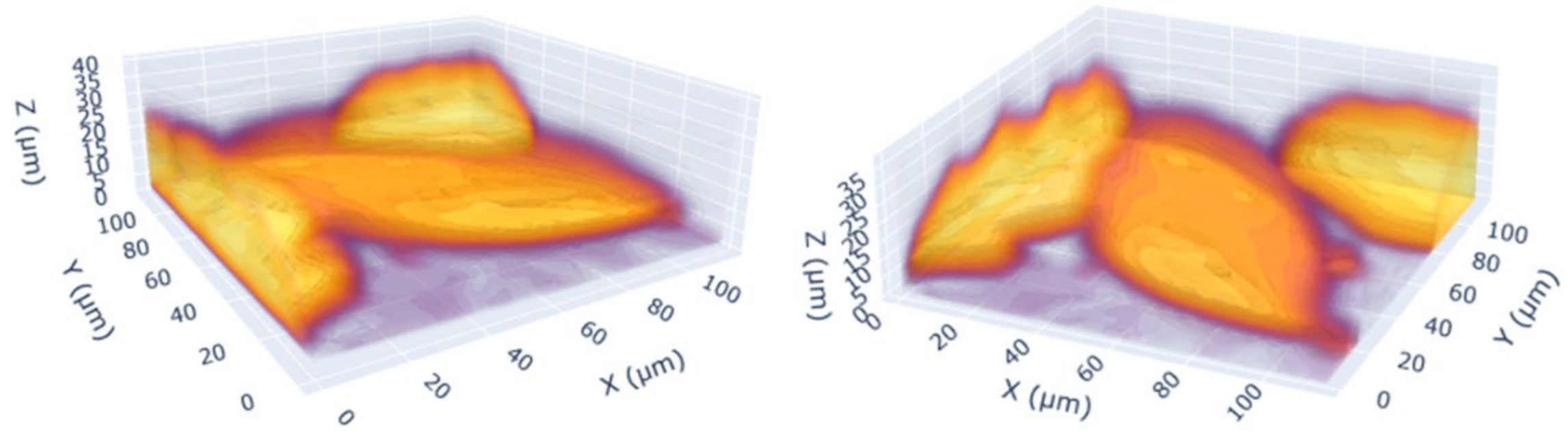

Figure S10: 3D luminescence intensity map of $LaPO_4$:Eu LC structures. Plots show the normalized luminescence intensity collected throughout the full volume for two different viewing angles.

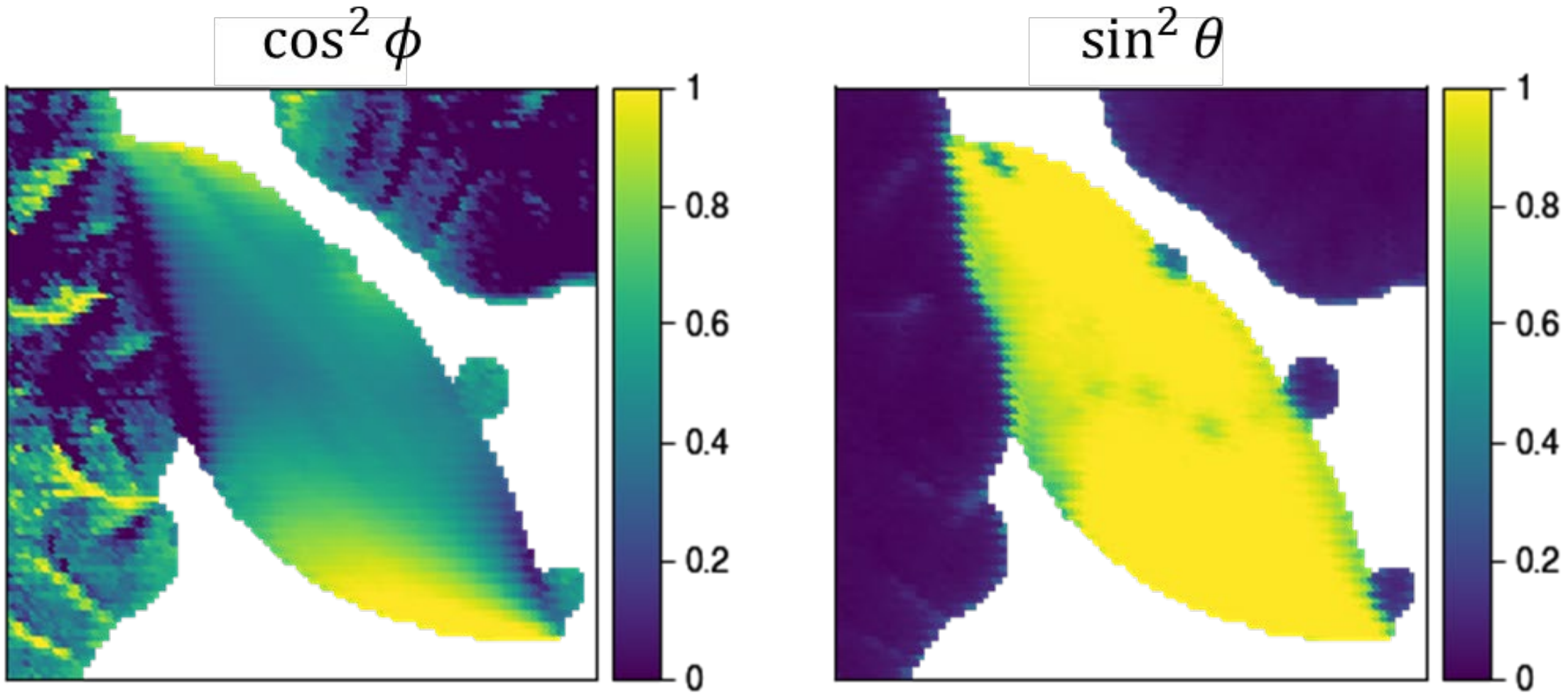

Figure S11: Tomographic maps of the results of spectral fitting the I_{zx} spectrum using equation 7 and 9.

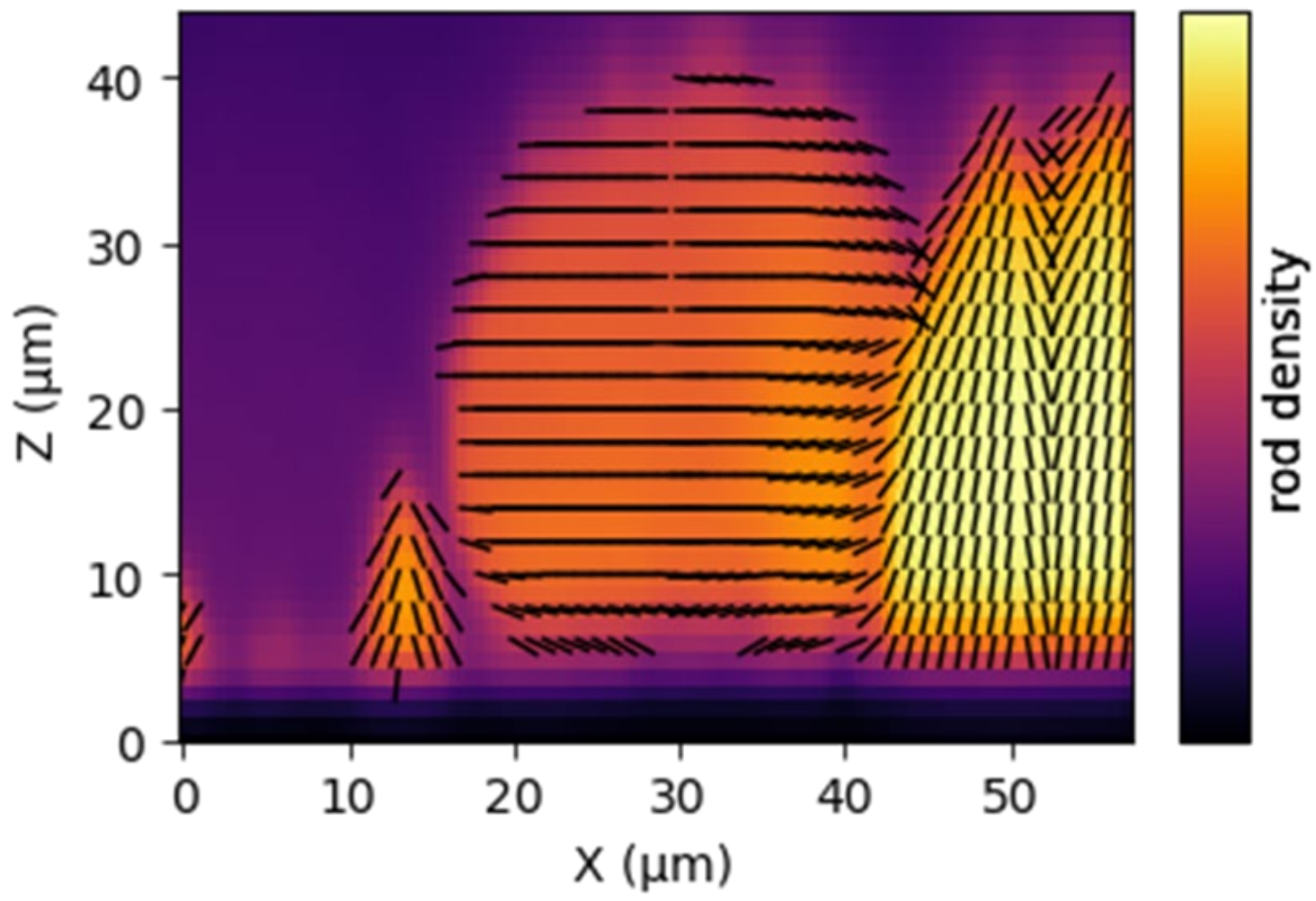

Figure S12: Cross section view of the orientation in a LC assembly. The director $\vec{n}$ is represented by the orientation of the bars and the background color is the normalized luminescence intensity, directly proportional to the rod density. The image corresponds to the x-z plane at the center y-coordinate of the plots in Figure 4-5.

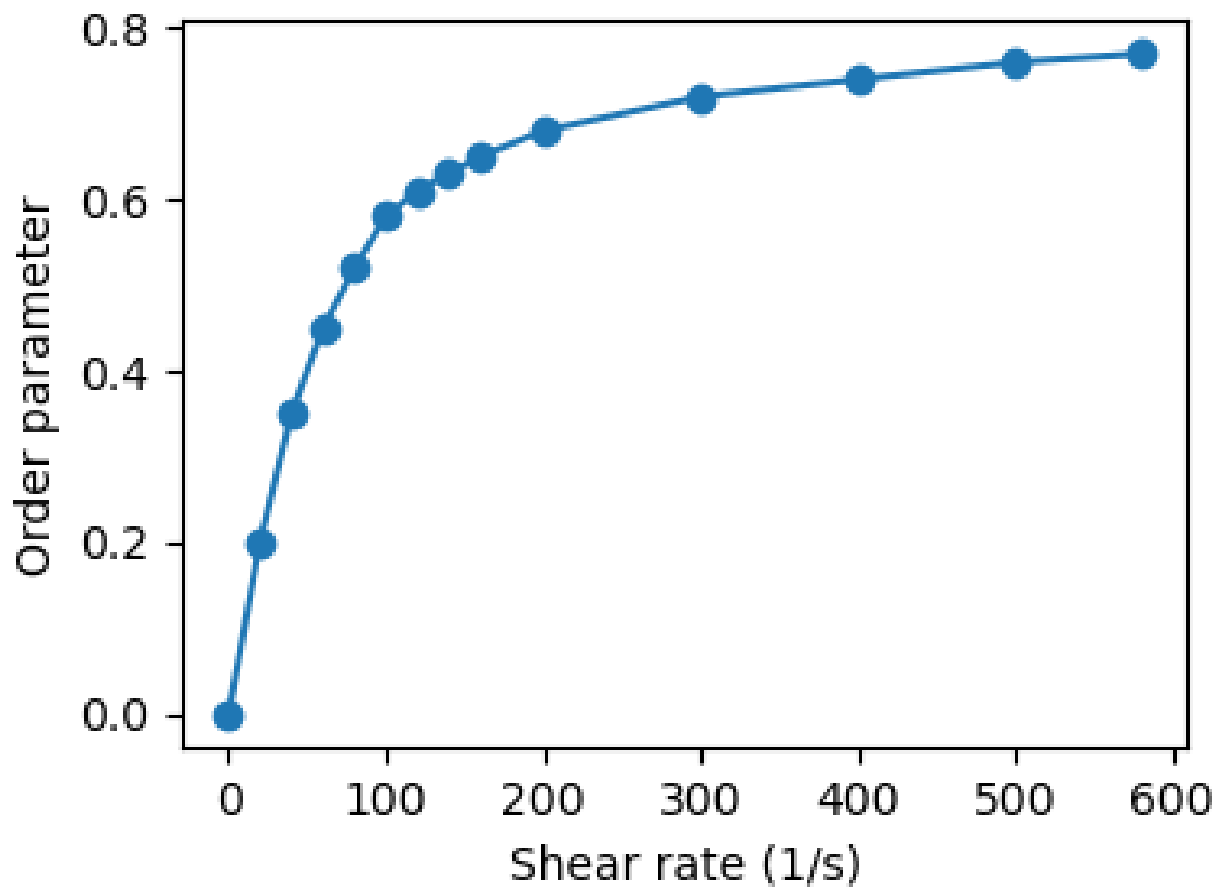

Figure S13: Calibration curve for an isotropic suspension of monazite $LaPO_4$:Eu nanorods in ethylene glycol. The order parameter is measured across a straight channel for known flow rates and plotted against the corresponding shear rate, as further detailed in our previous work.[6]

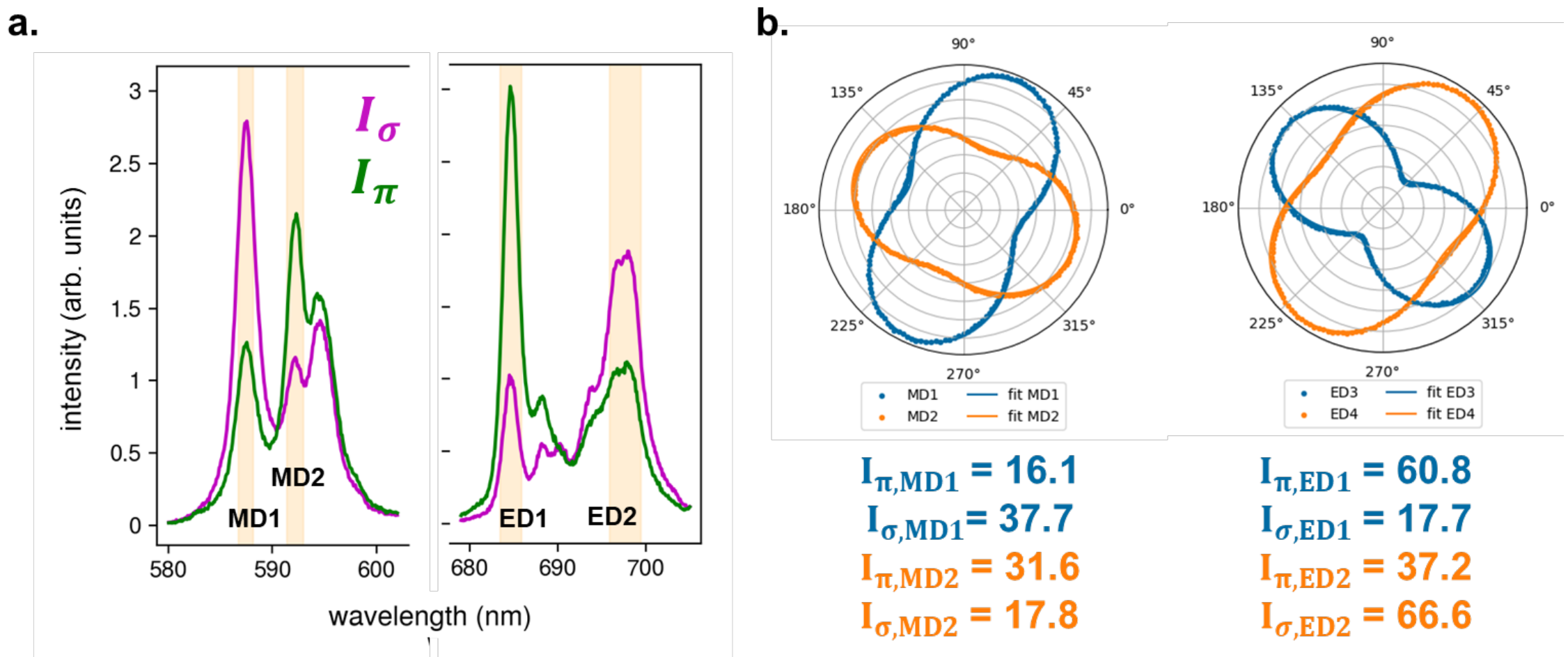

Figure S14: Reference spectra for monazite phase $LaPO_4$:Eu nanorods obtained in a planar LC assembly. a) The $I_\pi(\lambda)$ (green) and $I_\sigma(\lambda)$ (magenta) spectra. The highlighted regions correspond to the wavelength ranges used to calculate the area under the curve (AUC) used for orientation analysis. **b)** Angular dependency of the AUC of each subpeak when rotating the analyzer. The reference values labeled below the plots are found by fitting the data to the dipolar model (equation 3).

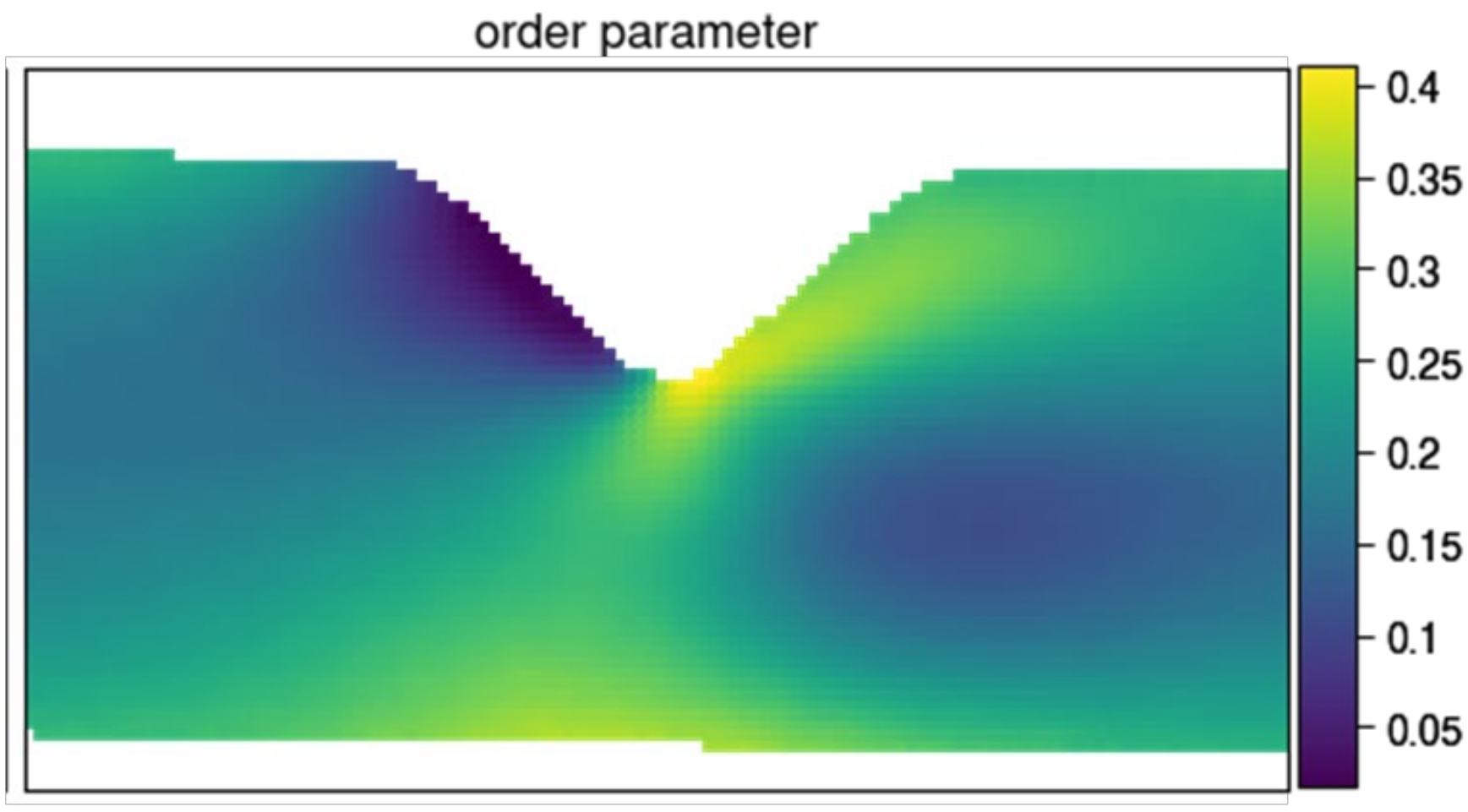

Figure S15: **Map of the order parameter S found through a simultaneous spectral fitting of I_{zx}, I_{z45° and I_{zy}.** The dataset matches that of Figure 6.

9. References

1. Kim, J., Lahlil, K., Gacoin, T. & Kim, J. Measuring the order parameter of vertically aligned nanorod assemblies. *Nanoscale* **13**, 7630–7637 (2021).
2. Goldstein, D. H. *Polarized Light*. (CRC Press, Boca Raton, 2017). doi:10.1201/b10436.
3. Menzel, M. *et al.* A Jones matrix formalism for simulating three-dimensional polarized light imaging of brain tissue. *Journal of The Royal Society Interface* **12**, 20150734 (2015).
4. Scheraga, H. A., Edsall, J. T. & Gadd, J. O. Double Refraction of Flow: Numerical Evaluation of Extinction Angle and Birefringence as a Function of Velocity Gradient. *The Journal of Chemical Physics* **19**, 1101–1108 (1951).
5. Peterlin, A. & Stuart, H. A. Uber die Bestimmung der Grobe und Form, sowie der elektrischen, optischen und magnetischen Anisotropie von submikroskopischen Teilchen mit Hilfe der kunstlichen Doppelbrechung und der inneren Reibung. *Physik* **112**, (1939).
6. Wang, Z. *et al.* Shearmetry of Fluids with Tunable Rheology by Polarized Luminescence of Rare Earth-Doped Nanorods. *ACS Nano* https://doi.org/10.1021/acsnano.4c09493 (2024).
