# Supplementary material for: Orientation tomography of lanthanide-doped nanoprobes by polarization-resolved micro-spectroscopy
Source: Nat Commun. 2026 Oct 14;17:11601. doi: 10.1038/s41467-026-78323-0 (PMC13751601; doi:10.1038/s41467-026-78323-0)
Supplement: Supplementary file 3 [file 41467_2026_78323_MOESM3_ESM.pdf]

nature portfolio

Peer Review File

Orientation tomography of lanthanide-doped nanoprobes by polarization-resolved micro-spectroscopy

Corresponding Author: Professor Jongwook Kim

Parts of this Peer Review File have been redacted as indicated to remove third-party material.

This file contains all reviewer reports in order by version, followed by all author rebuttals in order by version.

Version 0:

Reviewer comments:

Reviewer #1

(Remarks to the Author)
In this work, the authors present a polarization-resolved micro-spectroscopy methodology based on lanthanide-doped nanorods to achieve three-dimensional (3D) orientation tomography in both dense liquid-crystalline assemblies and dilute microfluidic systems. By exploiting the unique electric- and magnetic-dipole (ED/MD) transitions of lanthanide ions, the work establishes a combined experimental and theoretical framework to extract the director and order parameter from spectroscopic measurements. The approach is technically sophisticated and addresses an important challenge in orientation-resolved characterization of anisotropic systems. The work demonstrates strong innovation, rigorous experimentation, and a complete theoretical framework, holding significant application value for soft matter and fluid dynamics research. With these strengths, the manuscript is well-suited for publication in Nature Communications after addressing the following minor revisions:
1. The article proposes comparing simulated isotropic spectra with experimentally measured ones to validate the quality of reference spectra (Fig. 1e), which is a valuable new approach. However, if the samples used to obtain both the reference spectra and the isotropic spectrum are from the same batch preparation, the independence of this comparison is questionable. Should the reference sample itself have a systematic error, both the simulated and measured spectra would be affected by the same bias, rendering this validation method ineffective.
2. In the microfluidics experiment section, a fixed flow rate of 10 nL/min was used. Were there any limitations explored regarding the flow rate range? Was the variation of the order parameter Swith different flow rates considered?
3. The resolution of some figures needs enhancement. For example, the resolution measurement plot in Figure 2d.
4. Minor language and formatting issues should be corrected. Some specific instances are listed below, but this is not an exhaustive list:
• Line 92: “analysis. .”
• Line 177: “a droplet of diluted nanorod suspension”
• Line 460: “This work builds on our previous demonstrations”

Reviewer #2

(Remarks to the Author)
This manuscript demonstrates that a polarization-controlled spectroscopic method using nanorods can provide 3D orientational information about nanorod assemblies and the shear forces in the fluids in which nanorods are located. The presented results in this manuscript are closely related to their earlier published works using polarization-controlled confocal microspectroscopy of lanthanide-doped nanorods. Demonstrated improvements in the instrumentation include extending the acquisition time to accommodate emitters with long lifetimes, correcting chromatic aberration caused by large Stokes shifts, and mitigating polarization distortion due to various optical components.

Major comments:
The main novelty claimed by the authors is that they can determine the 3D orientation of nanorods by measuring the polarization profile of PL spectra without tilting the sample. However, as the authors note in the manuscript, the 3D angle of the collective nanorods cannot be determined without the knowledge of the order parameter of the orientational distribution.

Based on their analysis method, the order parameter can be determined only when the mean orientation of the nanorods is known to be either parallel or perpendicular to the beam direction. Unfortunately, the order parameter will likely change as the mean orientation angle deviates from the parallel or perpendicular direction. Thus, I feel that the main claim of “quantifying 3D orientation” is not sufficiently supported by the results presented in the manuscript. This makes this paper remain as a marginal updated version of their previous papers and be considered insufficiently novel for publication on Nature Communications.

Minor comments:
- Please provide description how the polarization state was calibrated. For example, specify if the half-waveplate is achromatic or zero-order. Also, typically, the extinction ratio of the 90-deg reflected beam from a polarization beamsplitter is lower than that of the transmitted beam.
- When the sample is thin, the polarization rotation by a birefringent medium is disregarded. However, the 60-um thick liquid crystal sample may rotate the PL Polarization before it exits the birefringent medium. Provide comments on this potentially significant effect on the data interpretation.
- When a reflecting metal surface is coated with a dielectric coating for protection, the polarization state of the reflected light can be distorted. However, unprotected (or bare) metal mirrors do not distort the polarization state of light. Some optics manufacturers provide unprotected metal mirrors for that purpose. The probable reason for the prism mirror showing less polarization distortion is that the prism's metal is unprotected by a dielectric coating.
- On p.13, “It should be noted that the technique is not sensitive to the polarization of the excitation beam, as for lanthanides the polarization of the emission is decoupled from the excitation.” This is uncommon with typical anisotropic emitters. Can the authors provide the reason why this nanorods show this unique behavior and references to support it?

Version 1:

Reviewer comments:

Reviewer #1

(Remarks to the Author)
The authors have addressed all the concerns that I have. I would suggest considering the acceptance of revised manuscript.

Reviewer #2

(Remarks to the Author)
The authors have satisfactorily addressed all minor comments.

In evaluating this manuscript for Nature Communications, I initially weighed two primary reservations. The first was regarding the breadth of its impact, as the methodology is restricted to determining the 3D orientation angle of lanthanide-doped nanocrystals. However, given the extensive use of these nanocrystals in diverse fields—ranging from advanced materials to biosciences—I agree that characterizing their orientation is of significant value and offers broad utility.

My second reservation involved the claims of "quantifying 3D orientation" and "3D orientation tomography." The proposed method relies on a separate measurement of a well-defined system; however, I acknowledge that this is an inherent physical limitation of single-viewing-angle measurements rather than a flaw in the study. The authors have successfully navigated this limitation with a robust theoretical framework and high-quality experimental execution.

Given these considerations, I find the revised manuscript to be scientifically sound and recommend it for publication.

Open Access This Peer Review File is licensed under a Creative Commons Attribution 4.0 International License, which permits use, sharing, adaptation, distribution and reproduction in any medium or format, as long as you give appropriate credit to the original author(s) and the source, provide a link to the Creative Commons license, and indicate if changes were made.
In cases where reviewers are anonymous, credit should be given to 'Anonymous Referee' and the source.
The images or other third party material in this Peer Review File are included in the article's Creative Commons license, unless indicated otherwise in a credit line to the material. If material is not included in the article's Creative Commons license and your intended use is not permitted by statutory regulation or exceeds the permitted use, you will need to obtain permission directly from the copyright holder.
To view a copy of this license, visit https://creativecommons.org/licenses/by/4.0/

Response to the reviewers

"Orientation tomography of lanthanide-doped nanoprobes by polarization-resolved micro-spectroscopy"

We thank the Reviewers for their careful evaluation of our manuscript and for their constructive and insightful comments. We have revised the manuscript accordingly and believe that the changes have improved its clarity and scientific quality. Below, we provide a point-by-point response to all comments, with the original comment in blue followed by the response in black. Changes in the revised manuscript are indicated in **blue**.

Reviewer #1

In this work, the authors present a polarization-resolved micro-spectroscopy methodology based on lanthanide-doped nanorods to achieve three-dimensional (3D) orientation tomography in both dense liquid-crystalline assemblies and dilute microfluidic systems. By exploiting the unique electric- and magnetic-dipole (ED/MD) transitions of lanthanide ions, the work establishes a combined experimental and theoretical framework to extract the director and order parameter from spectroscopic measurements. The approach is technically sophisticated and addresses an important challenge in orientation-resolved characterization of anisotropic systems. The work demonstrates strong innovation, rigorous experimentation, and a complete theoretical framework, holding significant application value for soft matter and fluid dynamics research. With these strengths, the manuscript is well-suited for publication in Nature Communications after addressing the following minor revisions:

We thank the reviewer for the careful review, insightful comments, and appreciation of our work.

Question 1

The article proposes comparing simulated isotropic spectra with experimentally measured ones to validate the quality of reference spectra (Fig. 1e), which is a valuable new approach. However, if the samples used to obtain both the reference spectra and the isotropic spectrum are from the same batch preparation, the independence of this comparison is questionable. Should the reference sample itself have a systematic error, both the simulated and measured spectra would be affected by the same bias, rendering this validation method ineffective.

The goal of this evaluation is to check if the collected reference spectra are purely σ and π polarization components without contamination by the axial emission due to an out-of-plane director tilt, which results in mixing of both σ and π. The proposed comparison of simulated and experimentally obtained isotropic spectra is an effective method for this evaluation. And for this, the reference spectra and the experimental isotropic spectra should be taken from the same batch if the emission properties of different batches are not perfectly identical. In practice, we have found that different batches could exhibit slightly different relative peak intensities due to variations in the crystallinity and defects generated during the nanorod synthesis. We are therefore systematically collecting new reference spectra when changing the batch of nanorods for the orientation analysis. To clarify this point, we have updated the corresponding sentences in the main text (lines 170-175).

We also conducted a supplementary simulation validating this approach. Figure R1 below illustrates the impact of a director tilt in the reference sample on the collected reference spectra and simulated isotropic spectrum. We clearly see that the tilt angle impacts the simulated isotropic spectrum. We have added several sentences in the main text and Figure R1 in the S.I. to emphasize this (lines 187-191).

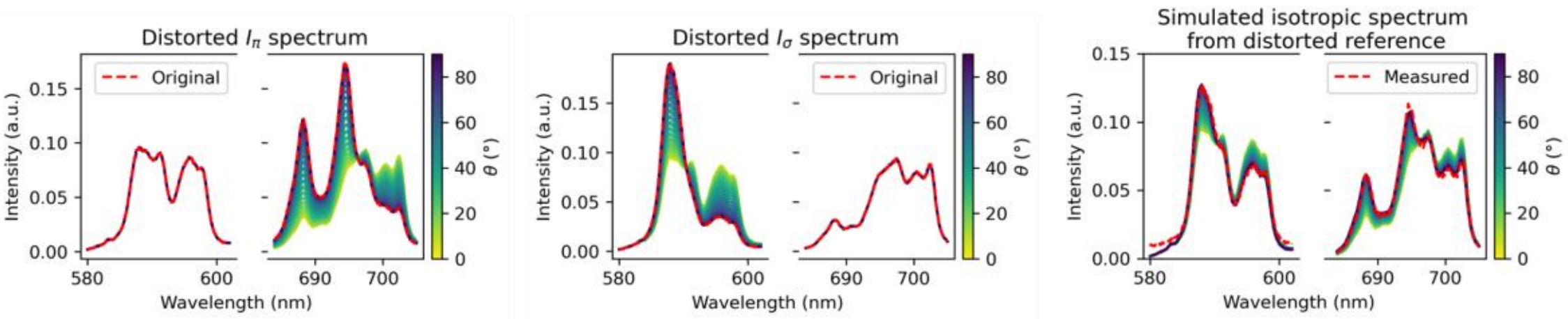

Figure R1: Simulated impact of the director tilt θ of the reference sample on the obtained reference spectra I_π , I_σ and simulated isotropic spectrum I_{iso} . The red dashed line represents the original experimentally measured I_π , I_σ, and I_{iso} respectively.

Question 2

In the microfluidics experiment section, a fixed flow rate of 10 nL/min was used. Were there any limitations explored regarding the flow rate range? Was the variation of the order parameter S with different flow rates considered?

The relationship between the order parameter (S) and the shear stress (τ, depending on the flow conditions including the flow rate) was established through a calibration procedure using a straight channel and various flow rates. A detailed study focusing on the fluidics and shearmetry applications is described separately in our recent report (reference #23). To keep the focus of this work on the optical methodology for orientation mapping, we selected one representative (10 nL/min) flow rate and show a corresponding order parameter and director map. To clarify this point, we added a description in the legend of figure 6 (line 422) and added the calibration curve figure in the S.I. (Figure S13).

Question 3

The resolution of some figures needs enhancement. For example, the resolution measurement plot in Figure 2d.

There was an image compression issue in our previous manuscript file. We fixed it in our revised manuscript.

Question 4

Minor language and formatting issues should be corrected. Some specific instances are listed below, but this is not an exhaustive list:
- Line 92: "analysis. ."
- Line 177: "a droplet of diluted nanorod suspension"
- Line 460: "This work builds on our previous demonstrations"

We have revised the entire manuscript following your advice. Thank you.

Reviewer #2:

Major comments:

This manuscript demonstrates that a polarization-controlled spectroscopic method using nanorods can provide 3D orientational information about nanorod assemblies and the shear forces in the fluids in which nanorods are located. The presented results in this manuscript are closely related to their earlier published works using polarization-controlled confocal microspectroscopy of lanthanide-doped nanorods. Demonstrated improvements in the instrumentation include extending the acquisition time to accommodate emitters with long lifetimes, correcting chromatic aberration caused by large Stokes shifts, and mitigating polarization distortion due to various optical components.

The main novelty claimed by the authors is that they can determine the 3D orientation of nanorods by measuring the polarization profile of PL spectra without tilting the sample. However, as the authors note in the manuscript, the 3D angle of the collective nanorods cannot be determined without the knowledge of the order parameter of the orientational distribution. Based on their analysis method, the order parameter can be determined only when the mean orientation of the nanorods is known to be either parallel or perpendicular to the beam direction. Unfortunately, the order parameter will likely change as the mean orientation angle deviates from the parallel or perpendicular direction. Thus, I feel that the main claim of "quantifying 3D orientation" is not sufficiently supported by the results presented in the manuscript. This makes this paper remain as a marginal updated version of their previous papers and be considered insufficiently novel for publication on Nature Communications.

We thank the reviewer for the careful review highlighting the important challenges for which we provided solutions and for another issue that remains as a fundamental limitation.

However, we respectfully disagree with the overall assessment, as we believe that the novelty and significance of several key aspects of this work may have been underestimated. First, the ability to measure both the 3D director (**n**) and the order parameter (S) through a purely spectroscopic approach represents a significant advancement. Second, considering that most existing tomography methods rely on scanning along the angular dimension, we would like to emphasize the impact of our 3D analysis principle by observation through a single viewing angle (i.e. 'without tilting the sample'). This approach offers important advantages in terms of adaptability and compatibility with conventional microscope platforms.

Furthermore, our comprehensive demonstration of both the theoretical framework and the technical solutions addressing the non-trivial challenges associated with the polarization of lanthanide emission provides a practical roadmap for reproducing this methodology and extending it to various research fields. We believe that the assessment of the 1st reviewer appropriately capture these multifaceted strengths and broad impact of our work as they state "*The work demonstrates strong innovation, rigorous experimentation, and a complete theoretical framework, holding significant application value for soft matter and fluid dynamics research. With these strengths, the manuscript is well-suited for publication in Nature Communications*".

The limitation pointed out by the reviewer – **n** and S cannot be determined when both are completely unknown – is indeed a fundamental problem inherent to any approach using a single viewing angle. Whereas other works in the literature either assume a perfect order (S=1) or a fully planar or vertical alignment to resolve the system, we provide a strategy to overcome this limitation on each casework by incorporating an additional observation or exploiting boundary conditions derived from the known physical properties of the sample. For the liquid crystal system, instead of simply assuming S=1, we directly measure S in a region where the tilt angle is known (vertical in this case) and apply this value for the subsequent orientation analysis. For the microfluidic system, we calculate the expected tilt angle profile in the cross section and show that incorporating this information significantly improves the accuracy of the reconstructed S profile (see Figure 7). We carefully describe this rational procedure for

constructing complete 3D maps throughout the manuscript, which further represents a significant progress over the previous works.

While this work builds on our previous publications, each of those studies focused on a distinct aspect that is independent of the major advances introduced in the present manuscript: Photophysics of lanthanide-doped nanocrystals [ref #26,27], Chemistry of nanocrystal synthesis [ref #19,24], Single particle orientation analysis [ref #1], Microfluidic shearmetry applications [ref #22,23,24], Physics of liquid crystal morphogenesis [ref #20, we newly added this reference which was just published on June 30th]. In contrast, the present work establishes a complete methodological framework that is broadly applicable to any system of lanthanide doped nanocrystals or molecular complexes. Furthermore, the comprehensive theoretical framework presented here, together with the detailed optical methodology tailored to the specific requirements of polarized lanthanide spectroscopy, constitutes a significant advance over our previous studies and provides a valuable resource for the broad readership of Nature Communications.

Minor comments:

Comment 1:

Please provide description how the polarization state was calibrated. For example, specify if the half-waveplate is achromatic or zero-order. Also, typically, the extinction ratio of the 90-deg reflected beam from a polarization beamsplitter is lower than that of the transmitted beam.

The waveplate is achromatic, and its orientation with respect to the laboratory frame was calibrated by analyzing polar diagrams of Eu^{3+} emission from reference samples (large monocrystalline $NaYF_4$:Eu microrods for which the rod axis can be determined directly on their micrograph images). The choice of the 90-deg reflected beam (s-polarized) from the polarizing beamsplitter (PBS) instead of the transmitted beam (p-polarized) was to minimize polarization-sensitive spectral distortion by the spectrometer gratings by matching the polarization parallel to the grooves. To filter out the remaining p-polarized light, we placed an additional linear polarizer between the PBS and the spectrometer. We forgot to incorporate this in the discussion of the experimental setup and have adjusted the experimental setup in Figure 2a and its discussion accordingly (lines 203-204).

Comment 2

When the sample is thin, the polarization rotation by a birefringent medium is disregarded. However, the 60-um thick liquid crystal sample may rotate the PL Polarization before it exits the birefringent medium. Provide comments on this potentially significant effect on the data interpretation.

We thank the reviewer for this insightful question. We didn't include this discussion in the original manuscript because this effect is minimal in the two specific cases demonstrated (as explained below). However, this can indeed cause a significant distortion especially when there exist multiple domains with different in-plane director angles (ϕ) or a continuous twist in ϕ across the sample thickness. We added a paragraph in the main text to address this issue (lines 360-365). We formulate here a method for correcting this, which has also been added to the SI.

We can model the nematic LC as an ideal linear retarder whose fast axis (parallel to the director) is defined as the x-axis such that its Jones matrix:

$$J = \begin{bmatrix} 1 & 0 \\ 0 & e^{i\delta} \end{bmatrix} \quad \text{(R1)}$$

where δ is the phase retardation as defined by:

$$\delta \approx \frac{2\pi}{\lambda} \cdot t \cdot \Delta n_{app} \tag{R2}$$

where λ is the wavelength of the illumination light, t is the sample thickness, Δn_{app} is the apparent birefringence and θ the obtained polar angle of the director [supplementary ref #1].

We then describe a linearly polarized PL emission component in terms of the angle between its polarization axis and the fast axis of the LC ψ such that:

$$E_{in} = \begin{bmatrix} \cos \psi_{in} \\ \sin \psi_{in} \end{bmatrix} \tag{R3}$$

The output then becomes

$$E_{out} = JE_{in} = \begin{bmatrix} \cos \psi_{in} \\ e^{i\delta} \sin \psi_{in} \end{bmatrix} \tag{R4}$$

For arbitrary retardance, the output is generally elliptically polarized. The orientation of the polarization ellipse is given by

$$\tan 2\psi_{out} = \frac{\sin 2\psi_{in} \cos\delta}{\cos 2\psi_{in}} \tag{R5}$$

Such that

$$\psi_{out} = \frac{1}{2} \text{atan2}(\sin 2\psi_{in} \cos \delta, \cos 2\psi_{in}) \tag{R6}$$

where $\text{atan2}(y, x)$ is the two-argument inverse tangent which returns the angle in the correct quadrant. From equation R6 we see that if ψ_{in} is 0 or a multiple of $\pi/2$, then $\Psi_{out} = \psi_{in}$. Therefore, if the polarization axis (equivalently the long axis of the polarization ellipse) of the propagating light is parallel or perpendicular to the fast axis of the LC, it's orientation will not be affected whilst propagating through the LC.

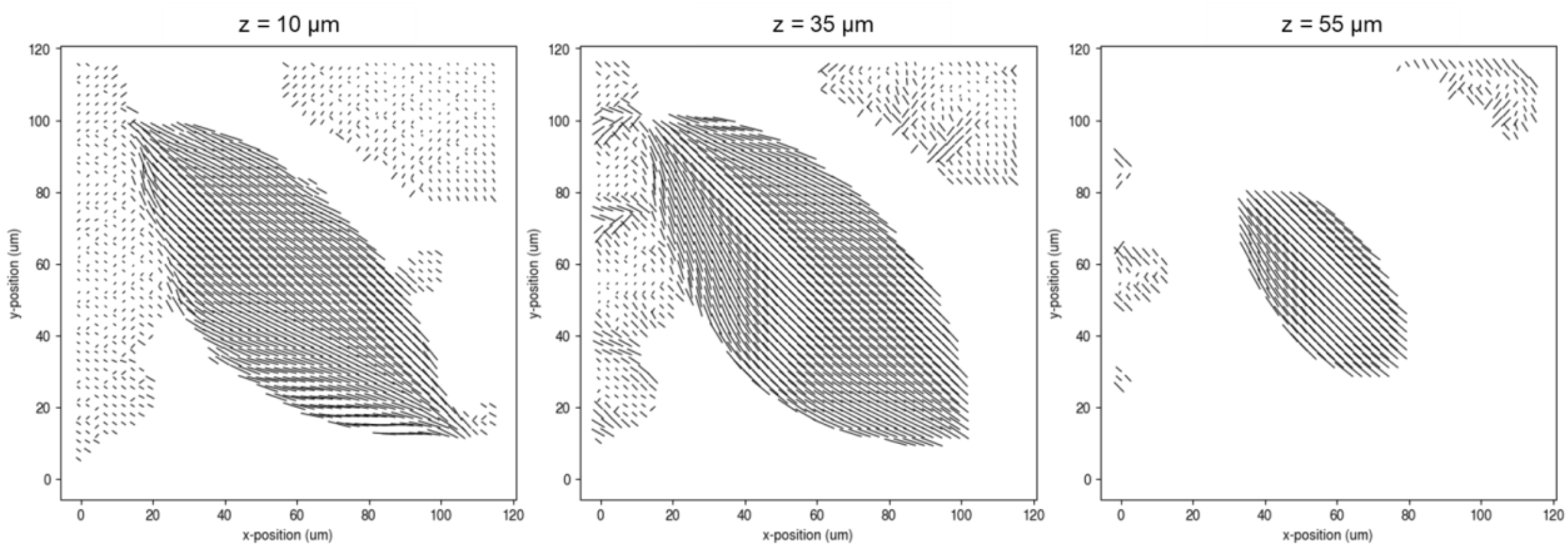

Figure R2: Director profile at 3 different xy planes corresponding to Figure 5e.

In the case of the lanthanide emission discussed here, the emission dipoles produce linearly polarized emission whose axes are parallel and perpendicular to the nanoparticles. Therefore, if the orientation of the nanorods is (close to) uniform throughout the depth of the sample, no rotation of the PL polarization

will be observed. This is indeed what we observed in the nematic LC samples. As shown in **Figure R2**, the measured director in the tactoid is largely uniform throughout the depth of the sample, and we thus do not observe any noticeable rotation in the polarization axis. In most colloidal LCs the twisting of the director (rotation across planes) is energetically unfavorable and we therefor do not expect significant apparent rotation of the polarization orientation measured with a linear analyzer.

However, when working with chiral (nematic) liquid crystals or other dense assemblies whose director field rotates within the observation plane through the depth of the sample, the effect of the birefringence can be substantial and should be taken into consideration.

Let us consider a measured director at a fixed x,y position throughout the depth of the sample described by the orientation $(\theta(z), \phi(z))$ as calculated by the spectroscopic approach presented in this work. We consider that z_0 is the bottom plane, such that no distortions due to birefringence affect its measurement and thus the correct azimuthal orientation would simply be $\phi_{corr}(z_0) = \phi(z_0)$. However, the measured orientation at the subsequent plane z_1 is affected by the PL emission propagating through plane z_0. The apparent birefringence of plane z_0 can be approximated as a function of the director orientation and order parameter S such that

$$\Delta n_{app,z_0} = \Delta n \cdot S \cdot \sin^2 \theta(z_0) \tag{R7}$$

where Δn is the intrinsic birefringence for perfectly aligned particles. For the $LaPO_4$:Eu LC discussed in this work, $\Delta n \approx 0.005$ [supplementary ref #2]. The retardance of plane z_0 can then be expressed using equation R2 such that

$$\delta_{z0} \approx \frac{2\pi}{\lambda_{PL}} \cdot (z_1 - z_0) \cdot \Delta n \cdot S \cdot \sin^2 \theta(z_0) \tag{R8}$$

The measured orientation $\phi(z_1)$ is affected by the birefringence of the underlying layer z_0. The polarization ellipse exits plane z_0 at a relative angle $\Psi_{out} = \phi(z_1) - \phi_{corr}(z_0)$. We want to find its original relative orientation Ψ_{in}, which can be done by rearranging equation R6 such that

$$\psi_{in} = \frac{1}{2}\text{atan2}\left(\frac{\sin 2\psi_{out}}{\cos\delta}, \cos 2\psi_{out}\right) \tag{R9}$$

Finally, we can convert the relative angle back to the laboratory frame by

$$\phi_{corr}(z_1) = \phi_{corr}(z_0) + \Psi_{in} \tag{R10}$$

This correction can then be applied throughout the whole volume by evaluating the expected distortion plane by plane from the bottom up (for an inverted microscope).

Comment 3

When a reflecting metal surface is coated with a dielectric coating for protection, the polarization state of the reflected light can be distorted. However, unprotected (or bare) metal mirrors do not distort the polarization state of light. Some optics manufacturers provide unprotected metal mirrors for that purpose. The probable reason for the prism mirror showing less polarization distortion is that the prism's metal is unprotected by a dielectric coating.

We have updated the main text to include this subtlety. We now use "conventional metal mirror" and mention that the distortion likely originates from the coating of the mirror (lines 259-261).

Comment 4

On p.13, "It should be noted that the technique is not sensitive to the polarization of the excitation beam, as for lanthanides the polarization of the emission is decoupled from the excitation." This is uncommon with typical anisotropic emitters. Can the authors provide the reason why this nanorods show this unique behavior and references to support it?

Lanthanide PL is characterized by energy transitions within the 4f orbitals, which are well shielded from the environment by the 5s and 5p shells. Coulombic interactions and spin-orbit coupling break the degeneracies of the 4f energy levels into discrete energy levels (**Figure R3**). These levels are then further split into sublevels by the electronic environment surrounding the lanthanide ion (the crystal field). This shielded crystal field splitting is the reason why the emission spectrum is composed of a number of sharp peaks that depend strongly on the host material.

[editorial note: third party material redacted]

Figure R3: The energy level splitting of the Eu^{3+} ion caused by various interactions. Figure from reference (Werts, 2005) [supplementary ref #3].

We use an indirect emission of Eu^{3+} by using a 394 nm laser for excitation, which corresponds to the $^7F_0$-$^5L_6$ transition. The excited electron then undergoes a significant energy loss by a non-radiative decay from the $^5L_6$ to the $^5D_0$ state. This decouples the absorption and emission, as the emission corresponds to transitions from the $^5D_0$ level to the ^7F_J levels. The polarization of the emission depends uniquely on the orientation of the emission dipole for each $^5D_0$-^7F_J transition, which are determined by the site symmetry of the lanthanide ion. We have added a clarifying sentence with citation to the main text (lines 269-271).

Although the polarization of the excitation laser may slightly impact the absorption cross section, and thus the overall emission intensity, this doesn't affect the degree of polarization of each emission peak. As our method considers relative peak intensities, excluding the effect of global intensity, the polarization of the excitation laser does not impact the orientation analysis.

Supplementary References

1. Goldstein, D. H. *Polarized Light.* (CRC Press, Boca Raton, 2017). doi:10.1201/b10436.
2. Kim, J. et al. Optimized combination of intrinsic and form birefringence in oriented LaPO4 nanorod assemblies. *Applied Physics Letters* **105**, 061102 (2014).
3. Werts, M. H. V. Making sense of Lanthanide Luminescence. *Science Progress* **88**, 101–131 (2005).
